# Asymmetric reduction of ketones and β-keto esters by (*S*)-1-phenylethanol dehydrogenase from denitrifying bacterium *Aromatoleum aromaticum*

**DOI:** 10.1007/s00253-014-6309-z

**Published:** 2014-12-31

**Authors:** A. Dudzik, W. Snoch, P. Borowiecki, J. Opalinska-Piskorz, M. Witko, J. Heider, M. Szaleniec

**Affiliations:** 1 Jerzy Haber Institute of Catalysis and Surface Chemistry, Polish Academy of Sciences, Niezapominajek 8, 30-239 Kraków, Poland; 2 Faculty of Chemical Engineering and Technology, Department of Biotechnology and Physical Chemistry, Cracow University of Technology, Warszawska 24 St., 31-155 Krakow, Poland; 3 Faculty of Chemistry, Warsaw University of Technology, Noakowskiego 3, 00-664 Warsaw, Poland; 4 Laboratory for Microbial Biochemistry, Philipps University of Marburg, Karl-von-Frisch Strasse 8, D-35043 Marburg, Germany

**Keywords:** Alcohol dehydrogenase, Optically pure alcohols, Hydrogen-transfer biocatalysis, Cofactor regeneration, Stereoselective bioreduction

## Abstract

Enzyme-catalyzed enantioselective reductions of ketones and keto esters have become popular for the production of homochiral building blocks which are valuable synthons for the preparation of biologically active compounds at industrial scale. Among many kinds of biocatalysts, dehydrogenases/reductases from various microorganisms have been used to prepare optically pure enantiomers from carbonyl compounds. (*S*)-1-phenylethanol dehydrogenase (PEDH) was found in the denitrifying bacterium *Aromatoleum aromaticum* (strain EbN1) and belongs to the short-chain dehydrogenase/reductase family. It catalyzes the stereospecific oxidation of (*S*)-1-phenylethanol to acetophenone during anaerobic ethylbenzene mineralization, but also the reverse reaction, i.e., NADH-dependent enantioselective reduction of acetophenone to (*S*)-1-phenylethanol. In this work, we present the application of PEDH for asymmetric reduction of 42 prochiral ketones and 11 β-keto esters to enantiopure secondary alcohols. The high enantioselectivity of the reaction is explained by docking experiments and analysis of the interaction and binding energies of the theoretical enzyme-substrate complexes leading to the respective (*S*)- or (*R*)-alcohols. The conversions were carried out in a batch reactor using *Escherichia coli* cells with heterologously produced PEDH as whole-cell catalysts and isopropanol as reaction solvent and cosubstrate for NADH recovery. Ketones were converted to the respective secondary alcohols with excellent enantiomeric excesses and high productivities. Moreover, the progress of product formation was studied for nine *para*-substituted acetophenone derivatives and described by neural network models, which allow to predict reactor behavior and provides insight on enzyme reactivity. Finally, equilibrium constants for conversion of these substrates were derived from the progress curves of the reactions. The obtained values matched very well with theoretical predictions.

**Electronic supplementary material:**

The online version of this article (doi:10.1007/s00253-014-6309-z) contains supplementary material, which is available to authorized users.

## Introduction

Enantiopure alcohols are one of the most valuable synthons for the production of various biologically active compounds such as pharmaceuticals, agrochemicals, or flavors (Kaluzna et al. 2005; Matsuda et al. 2009; Nakamura et al. 2003; Patel 2008, 2013). A straightforward approach to the synthesis of chiral alcohols is the asymmetric reduction of corresponding carbonyl compounds, which can be achieved by chemical or biocatalytic methods. Some well-established chemical methods are used for that purpose in industry, for example a variety of chiral metal complexes catalyzing asymmetric ketone reductions (Blaser et al. 2003; Ohkuma 2010). However, most of these methods need toxic metals and expensive metal hydrides, which require special reaction conditions. The alternative, more environmentally friendly approach to introduce chiral centers is available via biocatalytic methods.

Up to date, two general approaches have been commercialized to produce chiral alcohols: (i) kinetic resolution of racemic alcohols from esters by enantiospecific lipases (de Souza et al. 2009; Ghanem and Aboul-Enein 2005) or (ii) enantiospecific reduction of prochiral ketones by oxidoreductases (Breuer et al. 2004; Hasegawa et al. 2010; Kroutil et al. 2004; Nakamura et al. 2003; Patel 2008). The latter approach utilizes a fairly large and diverse group of enzymes that catalyze hydride transfer reactions in the presence of a coenzyme acting as hydride donor or acceptor. A number of different alcohol dehydrogenases (ADHs) have been utilized for asymmetrically reducing carbonyl functionalities. However, the wide spectrum of ADH isoenzymes, e.g., in regard to their substrate or enantiospecificities, still represents a major challenge in redox biocatalysis. Therefore, the search for enzymes better suited for a desired reaction needs to continue to further optimize the process.

Short-chain dehydrogenases/reductases (SDRs) are one of the largest enzyme superfamilies among the ADHs. They have received increasing attention due to their broad substrate specificities and tolerances against high temperatures and organic solvents (Li et al. 2013; Zhou et al. 2013), which are favorable characteristics of biocatalysts for chiral alcohol synthesis (Alsafadi and Paradisi 2013). SDRs are characterized by a length of ca. 250 amino acids, a Gly-rich motif in the coenzyme-binding regions and a catalytic triad formed in the active center by the highly conserved residues of a Tyr, Lys, and Ser, plus an Asn residue recently added according to Filling et al. (Tyr^154^, Lys^158^, Ser^141^, and Asn^113^ in PEDH) (Filling et al. 2002). The proposed mechanism of alcohol oxidation by SDRs implies an initial deprotonation of the tyrosyl residue, preceding substrate binding. The phenolate form of this group is proposed to participate in the binding of alcohol substrates together with the hydroxyl group of the Ser and to act as a nucleophilic catalyst for the hydride ion transfer from the CH group of the bound alcohol to NAD^+^ (McKinley-McKee et al. 1991). In reverting the reaction during ketone reduction, a hydride ion is formally transferred from NADH to the carbon atom of the carbonyl group, leading to an intermediary alkoxy (alcoholate) anion intermediate with concomitant proton transfer from the Tyr residue to the alcoholate. The Ser hydroxyl group seems to be essential in positioning the substrate via H-bond interactions, as its mutation to Ala or Phe renders SDRs inactive while its mutation to Tyr preserves enzyme activity (Filling et al. 2002; Jӧrnvall et al. 1995). The protons used in re-protonation of the active site tyrosine phenolate are retrieved from the solvent via a proton relay system composed of the conserved Lys and Asn residues together with the ribose hydroxyl groups of the NADH cofactor (Filling et al. 2002; Tanaka et al. 2001). As a result, the catalytic reactions of SDRs are strongly pH-dependent with pH optima of 8–9 for alcohol oxidation processes (proton needs to leave the active site) and around 5.5 for ketone reduction processes (proton needs to enter the active site) (Tanaka et al. 2001).

**Fig. 1:**
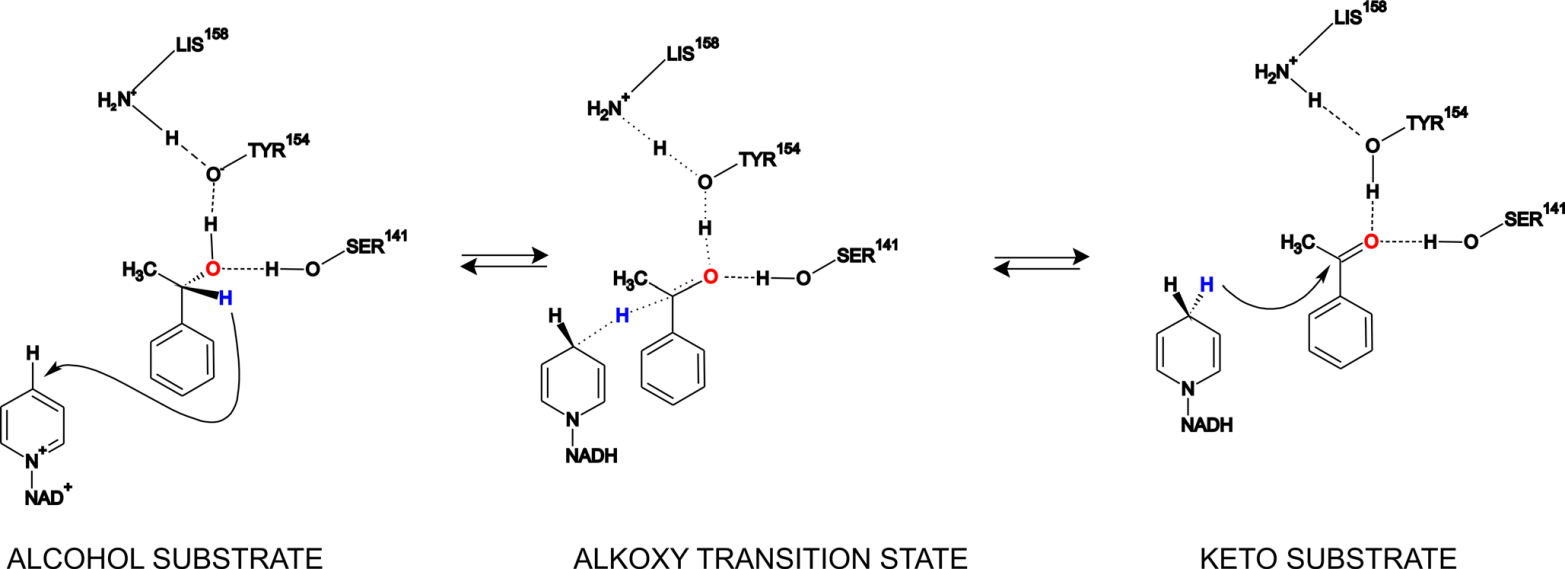
Reaction of alcohol oxidation and acetophenone reduction catalyzed by (*S*)-1-phenylethanol dehydrogenase

An enzyme of the SDR family, (*S*)-1-phenylethanol dehydrogenase (PEDH, EC 1.1.1.311), was found in the denitrifying bacterium *Aromatoleum aromaticum* (strain EbN1), which mineralizes ethylbenzene under anaerobic conditions (Rabus and Heider 1998; Rabus and Widdel 1995). Strain EbN1 produces (*S*)-1-phenylethanol via a direct anaerobic oxidation of ethylbenzene by the molybdenum enzyme ethylbenzene dehydrogenase (Kniemeyer and Heider 2001a; Szaleniec et al. 2007, 2014b). In the second step of the pathway, PEDH catalyzes the stereospecific conversion of (*S*)-1-phenylethanol to acetophenone and is also capable to catalyze the industrially important reverse reaction, namely, NADH-dependent enantioselective reduction of acetophenone to (*S*)-1-phenylethanol (Fig. 1) (Hӧffken et al. 2006; Kniemeyer and Heider 2001b).

In the present study, we investigated the substrate spectrum of PEDH, demonstrating that the enzyme reduces a wide range of compounds. The activity and enantioselectivity of PEDH were evaluated with a series of carbonyl compounds, especially aromatic and heterocyclic ketones and β-keto esters, demonstrating high enantioselectivity of the enzyme over the entire substrate range. The mechanistic basis of the high enantioselectivity of the reaction was evaluated by computer docking experiments and comparative analysis of interaction energies of theoretical enzyme-substrate (ES) complexes leading to *S*- and *R*-alcohols.

Moreover, we describe the progress of batch reactor tests with a whole-cell catalyst (resting *Escherichia coli* cells with the overexpressed recombinant PEDH), which was employed in reaction conditions similar to those used in industry (50- or 300-mM substrate concentrations). The reactions were carried out with an in situ NADH regeneration system utilizing sacrificial isopropanol (IPA) as a cosubstrate enabling reduction of NAD^+^ to NADH by the same enzyme (Goldberg et al. 2007), employing the suspended whole-cell catalyst in an IPA/water solvent. This makes the system very difficult to describe mathematically with standard equations for analytical reactors (Goudar et al. 2004; Nikolova et al. 2008). Therefore, we employed artificial neural network (ANN) modeling to determine the influence of various substrate parameters or reactor-starting conditions on the progress rates of product formation. ANN has been successfully applied to model various complex biological, medical, and chemical problems, especially where nonlinear relations are involved (Kuczkowski et al. 2004; Plawiak and Tadeusiewicz 2014; Szaleniec et al. 2013, 2014a; Tadeusiewicz 2011; Waligórski and Szaleniec 2010), and applications of ANN to predict enzyme reactivity or to model changes of reagents in the batch reactor have recently been reported (Abdul Rahman et al. 2009; Linko et al. 1999; Silva et al. 2008; Szaleniec 2012; Szaleniec et al. 2006).

## Materials and methods

### Preparation of the biocatalyst

PEDH was heterologously overexpressed in *E. coli* (strain TG1) containing the gene coding for PEDH from strain EbN1 behind a rhamnose-inducible promotor. Culture conditions and purification of the recombinant PEDH from *E. coli* were carried out as previously described (Hӧffken et al. 2006). Protein concentrations were measured according to the method of Bradford (1976).

### Enzyme assay

#### UV-Vis activity assay

Ketone reduction activity of purified PEDH was assayed at an optimum pH of 5.5 at 30 °C in 0.5-ml quartz cuvettes in 100-mM MES/KOH buffer containing 0.5 mM NADH and 5–10 μl of PEDH (app. 2 mg/ml). The assay was initiated by addition of the respective substrates from stock solutions in acetonitrile (end concentrations 0.5 mM), and NADH oxidation was followed at 365 nm (∆ε = 3.4*10^−3^ M^−1^ cm^−1^).

#### Synthesis of chiral alcohols with pure PEDH

The reaction mixtures were routinely conducted at 30 °C in 20 ml of 100-mM K_2_HPO_4_/KH_2_PO_4_ buffer (pH 5.8) containing 0.05 mM NADH and 100–200-μl purified PEDH (2 mg/ml). The reactions were initiated by addition of 100 μl of a respective substrate stock solution in IPA.The reactions were stopped after overnight incubation, and the analytes were extracted from the water phase by solid phase extraction using either C18 Polar Plus (Baker) or PS/DVB copolymer SPE columns (Strata-X from Phenomenex or the equivalent Chromabond HR-X from Macherey-Nagel), which were eluted with 0.5 ml of IPA. IPA extracts of reaction mixtures were directly analyzed by LC/MS.

#### Chromatographic analysis

The LC/MS analyses were performed on an Agilent 1100 System LC/MSD Quad VL equipped with a diode-array detector (DAD) and atmospheric pressure chemical ionization (APCI) in positive ion mode or electrospray (API-ES) in negative ion mode. The qualitative chiral analyses were performed using CHIRALCEL® OB-H column (Daicel, 250 × 4.6 mm, 5 μm, with a guard precolumn) in 25 °C and n-hexane/IPA as mobile phase at a flow rate of 0.5 ml/min with different isocratic programs, depending on the substance (see Table S1 of the supplementary material).

The n-hexane/IPA used in the normal phase chromatography is widely regarded as incompatible with the API ionization due to the high hazard of an n-hexane explosion upon contact with the heated nebulizer and high-voltage corona discharge (Alebic-Kolbah and Paul Zavitsanos 1997). Therefore, we used a 1:1 postcolumn addition of IPA/H_2_O/HCOONH_4_ according to previously established protocols (Alebic-Kolbah and Paul Zavitsanos 1997; Knack et al. 2013; Szaleniec et al. 2014b; Zavitsanos and Alebic-Kolbah 1998).

In most cases, the reaction enantioselectivity was determined based on the available standards of chiral products (aromatic ketones 1–23 and β-keto esters 43–46). The absolute configuration of 1-(4-methoxyphenyl) ethanol product was identified based on QSERR model (Szaleniec et al. 2009).

For the rest of the aromatic ketones and β-keto esters (24–42 and 47–53, respectively), either racemic standards of alcohol product were available or information about Chiralcel OB-H applications (Banoglu and Duffel 1997; Itoh et al. 2002; Kodama et al. 2012; Machado et al. 2009; Szaleniec 2012) allowed determination of enantiomeric excess of a major product fraction.

#### Batch reactor tests

Twenty-two batch reactor tests were performed at 30 °C in 50–100 ml of 60 % (*v*/*v*) solution of IPA and 40 % of 100-mM K_2_HPO_4_/KH_2_PO_4_ buffer (pH 5.8) containing 0.012-g/ml dry cell mass of resting *E. coli* cells with overproduced PEDH. Two types of batch reactors were evaluated with initial substrate concentrations of approximately 50 and 300 mM. During the reaction, 50-μl samples were collected, and the reaction was stopped by removing the bacterial cells via centrifugation (5 min at 15,000 RCF), followed by 100- or 200-fold dilution of the supernatant in acetonitrile. The samples were analyzed on reverse-phase mode HPLC using DAD as a detector. The quantitative HPLC data were used to generate reaction progress curves for both substrate and product. The chromatographic data were fitted by a mono-exponential function representing pseudo-first-order kinetics in the following form:

$$ \left[\mathrm{A}\right]\hbox{-} {\left[\mathrm{A}\right]}_{\mathrm{e}\mathrm{q}} = \left({\left[\mathrm{A}\right]}_0\hbox{-} {\left[\mathrm{A}\right]}_{\mathrm{e}\mathrm{q}}\right){\mathrm{e}}^{\hbox{-} \Big(\mathrm{k}}{1}^{+ \mathrm{k}}\hbox{-} {1}^{\Big)\mathrm{t}} $$

where [A] represents the concentration of a given substrate at time *t*, [A]_0_ its initial concentration, and [A]_eq_ its equilibrium concentration while k_1_ and k_-1_ represent the kinetic rate constants.

### Organic synthesis of standards

Most of the chiral standards of alcohol products were commercially available (see supplementary material for a list of compounds, their purities, and producers, Table S2). Custom synthesis of the enantiomerically pure alcohols (*S*)-1-(4-ethylphenyl)ethanol, (1*S*,1′*S*)-1-(4-(1-hydroxy-ethyl)-phenyl)-ethanol, (*S*)-1-(3-methylphenyl)ethanoland (*S*)-1-([1,1′-biphenyl]-4-yl)ethanol, and for the racemic compounds 1-(4-ethylphenyl)ethanol, (1*S*,1′S)-1,1′-(benzene-1,4-diyl)diethanol, and 1-(3-methylphenyl)ethanol was conducted according to the previously established protocols (Szaleniec et al. 2009, 2014b). Synthesis of the racemic and chiral standards of 3-aryl-3-hydroxypropionates was described in Borowiecki and Bretner (2013).

### Thermochemical calculations

Estimation of reaction equilibrium constant (K = e^(−ΔG/RT)^, where K = [acetone][product]/[IPA][substrate]) was based on theoretical calculation of G for individual reactants (acetone, IPA, alcohol product, ketone substrate, assuming concentration of all reactants as 1 M). The influence of substituents on stabilization of the alkoxy anion was estimated by calculation of ∆G for alkoxy anion formation (ketone + H^−^ = alkoxy anion). All calculations were conducted in vacuo in Gaussian 09 (Frisch et al. 2009) on the B3LYP/6-31G(d, p) level of theory similarly to the previously established protocol (Szaleniec et al. 2008). Thermochemical descriptors (∆G or reaction and ∆G of alkoxy anion formation) were established for nine selected *p*-substituted acetophenone derivatives which exhibited a wide range of electro-donor effect introduced by the *p*-substituent (Hammett σ range of −0.37 to + 0.78). The values obtained for each of the compounds were related to ΔG of alkoxy anion formation of acetophenone, thus generating ΔΔG^alkoxy^ values relative to the reduction of acetophenone. The obtained values correlated excellently with the Hammett parameters (*R* ^2^ = 0.94; see supplementary material, Fig. S1). Moreover, ΔΔG^alkoxy^ values can also be calculated for derivatives with unknown σ_p_ parameters. The theoretical values of logK of several PEDH substrates were correlated with experimentally obtained log K^ex^ values estimated from the batch reactor tests of the same substrates.

### Artificial neural networks

Development of ANN architecture as well as model training and validation was conducted with Neural Networks module of Statistica 7.1 (www.statsoft.com). The data set comprised cases which collected data on reaction of acetophenone and its eight derivatives with different *para*-substituents (*p*-Cl, *p*-Br, *p*-F, *p*-C_2_H_5_, *p*-OCH_3_, *p*-OH, *p*-methanesulfonyl, *p*-NO_2_) in the aromatic ring. Each case comprised reaction time and concentration of the product. The substrates were additionally characterized by Hammett σ_p_, ∆∆G of alkoxy anion formation, log K, log P, molecular refractivity MR (calculated in the Accerlys Discovery Studio 4.0, see Table S3 of the supplementary material) as well as initial substrate concentration (approximately 50 or 300 mM). To investigate the influence of electron donor-acceptor effect on the progress of reduction, we selected substrates with a wide σ_p_ range of −0.37 to +0.78. However, as the σ_p_ is available only for a limited range of substrates, we decided to evaluate ΔΔG^alkoxy^ descriptor as a convenient substitute. In order to estimate the substrates, influence on the final conversion log K was used. It turned out that the molecular modifications which stabilize formation of alkoxy anion stabilize formation of alcohols. As a result, there was a strong linear correlation between ΔΔG^alkoxy^ and log K (*R* = 0.93) as well as Hammett’s and log K (*R* = 0.96) (see Fig. S1 of the supplementary material). Therefore, it was not possible to separately determine the influence of kinetic and thermodynamic effects on the observed reactor progress curves. The enzyme reactivity can also depend on the efficacy of substrate fit to active site, i.e., substrate size and hydrophobicity (Naik et al. 2012). Therefore, simple parameters like MR and octanol-water partition coefficiency (log P) describing substrate size and hydrophobicity were introduced to our modeling.

The neural models used concentration of products at a given time as a dependent variable which were randomly divided into training, validation, and test subsets in ratio 2:1:1. Details of the experimental data processing are available in the supplementary materials.

The initial removal of the redundant variables was optimized by Intelligent Problem Solver (IPS), a heuristic algorithm implemented in SNN based on the validation error. This process resulted in the elimination of MR and Log P. These descriptors were not considered in further model optimization. Highly correlated variables, σ_p_ Hammett, ∆∆G of alkoxy anion formation, and log K (*R* ^2^ in the range of 0.85–0.94), cannot be used as linearly independent variables. Therefore, the models were developed with only one of this three descriptors at a time. The architecture of neural networks was obtained by IPS, which selects best type of neural network, best input vector, optimal number of hidden neurons, as well as the best type of activation function on output neuron based on minimal error for validation set. For each type of networks, 500 of linear or multiple layer perceptron (MLP) neural networks were tested by ISP with universal training techniques, and best 3 networks exhibiting lowest prediction error in validation subset were chosen for manual training and analysis. These models were subsequently manually retained with combination of training algorithms implemented in Statistica 7.1. The postprocessing sensitivity analysis and response analysis were used to determine the importance of input variables and their influence on the predicted product concentration. The final robustness of the obtained models was evaluated based on quality of predictions for external validation set which comprised four experiments (conversion of 52, 47, 107, and 356 mM 4′-fluoroacetophenone (**10**)). For this reason, data for conversions of **10** were excluded from the set during the establishment of the model and were used later for external validation.

### Substrate docking and calculation of interaction energies

All calculations were conducted with Discovery Studio 4.0 using general-purpose ChARMm force field with Momany-Rone partial charges (Momany and Rone 1992). The model of the enzyme based on the crystallographic structure of the PEDH:NAD^+^ complex (PDB 2EWM) where NAD^+^ was modified to NADH and only one subunit (chain B) with complete polypeptide chain was used. Prior to the docking, solvent molecules were removed, and the residues of the active site (5 Å radius from NADH) were minimized with 0.004-kJ/mol gradient tolerance using a distance-dependent dielectric model solvent. Acetophenone and its substituted derivatives (**1**, **7**, **21**, **22**, **28**, **29**, **43**, and benzophenone) were docked into the active site using LigandFit protocol (Venkatachalam et al. 2003). The obtained poses were filtered for hydrogen bond interactions with Ser^141^ or Tyr^154^, and ligand poses that did not form any H-bonds with these residues were not considered in further analysis. The docking poses were scored with Libdock score (PLP like score comprised Steric and H-bonding intermolecular functions), where the higher PLP scores indicate stronger ligand binding affinity to the receptor (Suveena and Lilly 2011). Moreover, the obtained poses were analyzed in terms of the ligand face exposed toward NADH (either pro(*S*) or pro(*R*)). The geometries of the best scored pro(*S*) and pro(*R*) docking poses were minimized (substrate, NADH, and all residues in 12 Å radius from OH group of Tyr^154^ using a smart minimizer algorithm with RMS gradient of 0.004 kJ/mol) using a distant-dependent dielectric solvent model (ε = 4). Finally, the binding energies and binding entropies of the substrates were estimated according to the method developed by Tirado-Rives et al. (Tirado-Rives and Jorgensen 2006) using the same reference geometry as for the enzyme without substrate (with active site residues minimized in 12 Å radius from the Tyr^154^ OH group). The interaction energies (sum of vdW and electrostatic interaction energies partitioned per residue) were calculated between the docked substrates, 65 amino acids (those within 12 Å from the Tyr^154^ OH group) and the NADH cofactor. The intermolecular interactions were analyzed and compared between different substrates and the theoretical pro(*S*) and pro(*R*) poses.

## Results

### Enzyme enantioselectivity

PEDH catalyzed the enantioselective reduction of a broad spectrum of carbonyl compounds: 42 ketones of different chain lengths with aromatic or heterocyclic substituents and the methyl esters of 11 aromatic β-keto acids with *meta* and *para* substituents in the aromatic ring were converted (Table 1). In most cases, the absolute configurations of the products were determined, and almost all investigated reactions yielded only one stereoisomer of the product (within detection limits). The exceptions were reduction of 4′-hydroxyacetophenone (**22**) which yielded 5 % of the second enantiomer (90 % ee % of major fraction) and of 4′-aminoacetophenone (**21**) which yielded the racemic mixture of 1-(4-aminophenyl)ethanol. A more detailed investigation of the reaction conditions for **21** showed that the reaction becomes stereospecific at higher pH values, and when the reaction was conducted at pH of 8.0, only one enantiomer was produced. This effect was attributed to an acid-induced racemization of the product rather than a pH-dependent enantioselectivity of the enzyme, because chirally pure 1-(4-aminophenyl)ethanol extracted with isopropanol was completely racemized after 24 h when the pH was decreased by addition of TCA (0.6 N).

Stereoselective reduction of carbonyl compounds by PEDH follows the Prelog rule (Prelog 1964). As a result, almost all of the recognized product enantiomers showed (*S*)-configuration except for compounds with reversed CIP priority (e.g., 2-chloro-1-phenylethanol) which were assigned with an (*R*)-configuration (**3**, **27**–**31**, **39**). This result is consistent with the previously proposed binding mode of PEDH substrates (Hӧffken et al. 2006) which was additionally confirmed by our docking studies (see below) (Table 1)

**Table 1:** Substrates converted by PEDH

No	Substrate	Product	S [%]	R [%]	%ee
Aromatic ketones					
1	acetophenone	1-phenylethanol	100	0	100
2	propiophenone	1-phenylpropan-1-ol	100	0	100
3	2-chloroacetophenone	2-chloro-1-phenylethanol	0	100	100
4	4′-ethylacetophenone	1-(4-ethylphenyl)ethanol	100	0	100
5	4′-acetyl acetophenone	1-(4-(1-hydroxy-ethyl)-phenyl)-ethanol	100	0	100
6	(*S*)-1-[4-(1-hydroxyethyl)phenyl]-methylketone	1-(4-(1-hydroxy-ethyl)-phenyl)-ethanol	100	0	100
7	4′-acetylbiphenyl	1-(biphenyl-4-yl)ethanol	100	0	100
8	4′-acetylphenyl methanesulfonate	4-(1-hydroxyethyl)phenyl methanesulfonate	100	0	100
9	4′-nitroacetophenone	1-(4-nitrophenyl)ethanol	100	0	100
10	4′-fluoroacetophenone	1-(4-fluorophenyl)ethanol	100	0	100
11	3′-hydroxyacetophenone	3-[(1)-1-hydroxyethyl]phenol	100	0	100
12	3′-methylacetophenone	1-(3-methylphenyl)ethanol	100	0	100
13	1-indanone	1-indanol	100	0	100
14	1-(furan-2-yl)methylketone	1-(furan-2-yl)ethanol	100	0	100
15	3-acetylpyridine	1-(pyridin-3-yl)ethanol	100	0	100
16	2-acetylthiophene	1-(thiophen-2-yl)ethanol	100	0	100
17	2-acetylpyridine	1-(pyridin-2-yl)ethanol	100	0	100
18	4-acetylpyridine	1-(pyridin-4-yl)ethanol	100	0	100
19	1-tetralone	1,2,3,4-tetrahydronaphthalen-1-ol	100	0	100
20	4′-methoxyacetophenone	1-(4-methoxyphenyl)ethanol	100	0	100
21	4′-aminoacetophenone	1-(4-aminophenyl)ethanol	50	50	0
22	4′-hydroxyacetophenone	4-(1-hydroxyethyl)phenol	95	5	90
23	2′-hydroxyacetophenone	2-(1-hydroxyethyl)phenol	100	0	100
24	(*S*)-3-phenyl-1-indanone	(*S*)-3-phenyl-1-indanol			100
25	6-hydroxy-1-indanone	2,3-dihydro-1*H*-indene-1,6-diol			100
26	2′-fluoroacetophenone	1-(2-fluorophenyl)ethanol			100
27	2,2-dichloroacetophenone	2,2-dichloro-1-phenylethanol			100
28	2,4′-dichloroacetophenone	2-chloro-1-(4-chloro-phenyl)-ethanol			100
29	2,2,2-trifluoroacetophenone	1-phenyl-2,2,2-trifluoroethan-1-ol			100
30	2,2-difluoroacetophenone	1-phenyl-2,2-trifluoroethan-1-ol			100
31	2-fluoroacetophenone	2-fluoro-1-phenylethan-1-ol			100
32	3′-aminoacetophenone	1-(3-aminophenyl)ethanol			100
33	4′-chloroacetophenone	1-(4-chlorophenyl)ethanol			100
34	4′-bromoacetophenone	1-(4-bromophenyl)ethanol			100
35	4-acetylbenzonitrile	4-(1-hydroxyethyl)benzonitrile			100
36	2-cyanoacetophenone	3-hydroxy-3-phenylpropanenitrile			100
37	3′-chloroacetophenone	1-(3-chlorophenyl)ethanol			100
38	3′-methoxyacetophenone	1-(3-methoxyphenyl)ethanol			100
39	3-coumaranone	2,3-dihydro-1-benzofuran-3-ol			100
40	3'-fluoroacetophenone	1-(3-fluorophenyl)ethanol			100
41	3'-bromoacetophenone	1-(3-bromophenyl)ethanol			100
42	(*R*)-3-phenyl-1-indanol	(*R*)-3-phenyl-1-indanol			100
β-keto esters					
43	methyl 4-fluorobenzoylacetate	methyl 3-(4-fluorophenyl)-3-hydroxypropanoate	100	0	100
44	methyl (4-chlorobenzoyl)acetate	methyl 3-(4-chlorophenyl)-3-hydroxypropanoate	100	0	100
45	methyl 4-bromobenzoylacetate	methyl 3-(4-bromophenyl)-3-hydroxypropanoate	100	0	100
46	3-(4-methoxy-phenyl)-3-oxo-propionic acid methyl ester	methyl 3-hydroxy-3-(4-methoxyphenyl)propanoate	100	0	100
47	3-(3-bromo-phenyl)-3-oxo-propionic acid methyl ester	methyl 3-(3-bromophenyl)-3-hydroxypropanoate			100
48	3-(3-methoxyphenyl)-3-oxo-propionic acid methylester	methyl 3-hydroxy-3-(3-methoxyphenyl)propanoate			100
49	3-(3-chloro-phenyl)-3-oxo-propionic acid methyl ester	methyl 3-(3-chlorophenyl)-3-hydroxypropanoate			100
50	3-(3-fluoro-phenyl)-3-oxo-propionic acid methyl ester	methyl 3-(3-fluorophenyl)-3-hydroxypropanoate			100
51	methyl 3-(4-methylphenyl)-3-oxopropanoate	methyl 3-hydroxy-3-(4-methylphenyl)propanoate			100
52	methyl 3-(4-ethylphenyl)-3-oxopropanoate	methyl 3-(4-ethylphenyl)-3-hydroxypropanoate			100
53	methyl 3-oxo-3-phenylpropanoate	methyl 3-hydroxy-3-phenylpropanoate			100

*S*/*R* [%] amount of isomers identified based on chiral standards and absolute configurations, %ee enantiomeric excess identified based of racemic standards or Chiralcel OB-H applications: 1-(3-chlorophenyl)ethanol (Kodama et al. 2012), 1-(3-methoxyphenyl)ethanol (Itoh et al. 2002), 2-chloro-1-(4-chloro-phenyl)-ethanol (Itoh et al. 2002), 1-(4-chlorophenyl)ethanol (Szaleniec 2012), 1-(3-fluorophenyl)ethanol (Banoglu and Duffel 1997), 1-(pyridin-3-yl)ethanol, and 1-(pyridin-4-yl)ethanol (Machado et al. 2009). Tests conducted with benzophenone (diphenylmethanone) showed no enzymatic activity

### Modeling of enzyme-substrate complexes

**Fig. 2:**
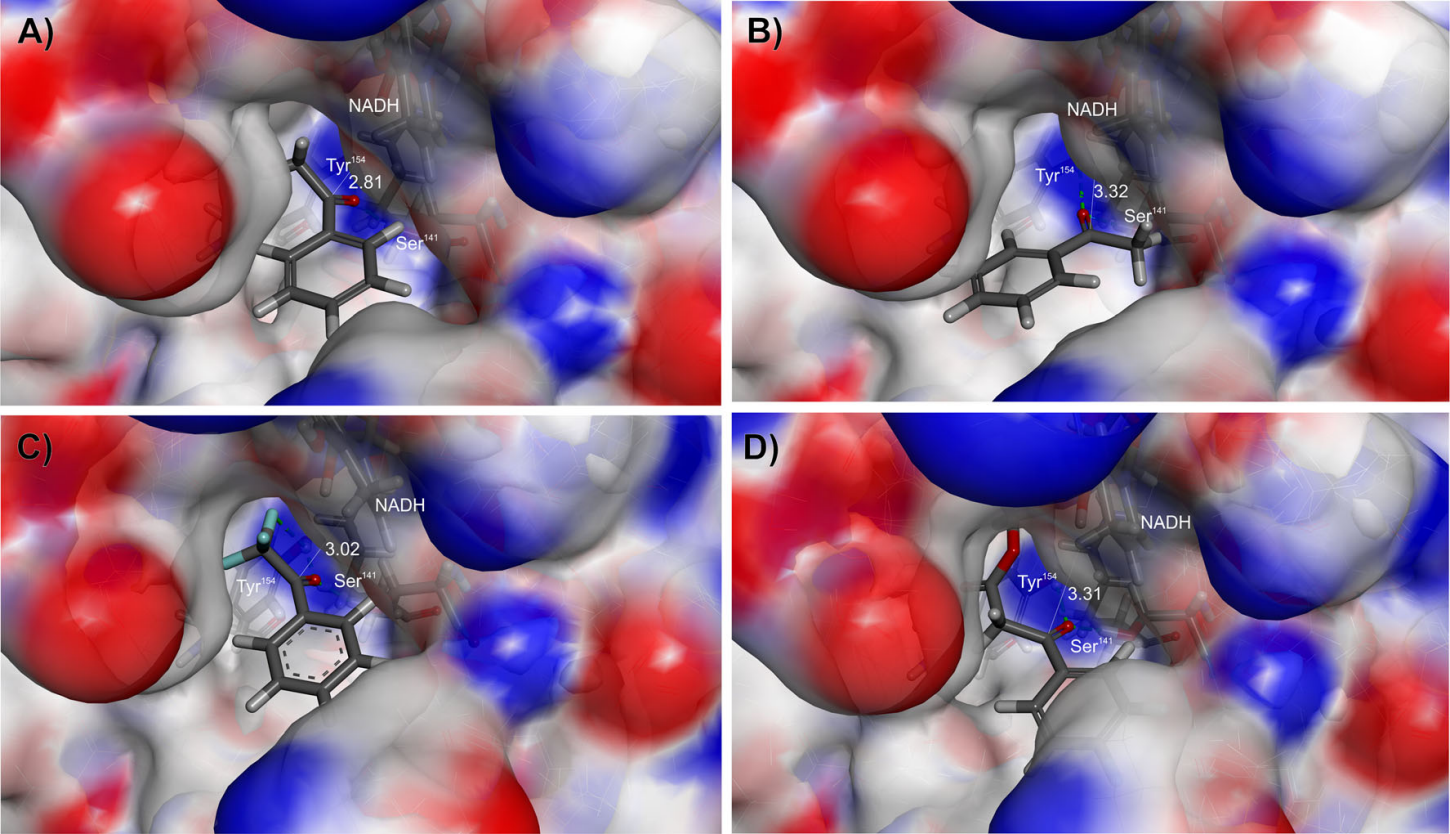
Models of PEDH-substrate-NADH ternary complexes: **a** Prelog orientation (Pro(*S*)) and **b** anti-Prelog (Pro(*R*)) orientation of acetophenone, **c** Prelog orientation (Pro(*R*)) of 2,2,2-trifluoroacetophenone (reversed CIP priority), and **d** Prelog (pro(S)) orientation of methyl 4-fluorobenzoylacetate. The vdW surface is colored with H-bond acceptor (*blue*)/H-bond donor (*red*) capabilities of the protein residues. The distances between the H-atom of NADH and the benzylic carbon atoms of the substrates are provided in Å

The structures of seven ES complexes with representative ligands (**1**, **7**, **21**, **22**, **28**, **29**, **43**) covering the structural diversity of the studied substrates were studied. For acetophenone (**1**), 4′-aminoacetophenone (**21**) and 4′-hydroxyacetophenone (**22**), both hypothetic Prelog and anti-Prelog complexes were modeled, whereas for 4′-acetylbiphenyl (**7**), 2,4′-dichloroacetophenone (**28**), 2,2,2-trifluoroacetophenone (**29**), and methyl 4-fluorobenzoylacetate (**43**), only Prelog complexes could be obtained from modeling (Fig. 2 and Fig. S2 of the supplementary materials). The binding energies were in the range of −192 to −100 kJ/mol for the Prelog complexes (−105 to −29 kJ/mol total binding energy (TBE) if binding entropy was accounted for), which were approximately 34 kJ/mol lower than those of the accessible anti-Prelog complexes. In addition, the calculated interaction energies of the Prelog conformations were 20–33 kJ/mol lower than those of the anti-Prelog conformations (Table 2), indicating the enzyme’s preference for substrate binding in Prelog conformation.

**Table 2:** Calculated pro(*S*) and pro(*R*) total interaction energy (TIE) (kJ/mol), binding energies (BE), and total binding energies corrected for binding entropy (TBE with ∆S) for PEDH-NADH-substrate ternary complexes

No.	Name	TIE (kJ/mol)		BE (kJ/mol)		TBE with ∆S (kJ/mol)	
		Pro(*S*)	Pro(*R*)	Pro(*S*)	Pro(*R*)	Pro(*S*)	Pro(*R*)
1	Acetophenone	-109.5	-91.2	-101.5	-78.3	-29.3	-6.2
7	4′-acetylbiphenyl	-138.1	n.d.	-117.6	n.d.	-36.0	n.d.
21	4′-aminoacetophenone	-128.0	-95.6	-116.1	-82.6	-42.7	-9.1
22	4′-hydroxyacetophenone	-129.1	-96.9	-118.3	-86.6	-44.7	-13.0
28	2,4′-dichloroacetophenone^a^	n.d.	-135.0	n.d.	-140.2	n.d.	-38.7
29	2,2,2-trifluoroacetophenone^a^	n.d.	-133.5	n.d.	-120.5	n.d.	-38.7
43	Methyl 4-fluorobenzoylacetate	-152.8	n.d.	-194.3	n.d.	-107.7	n.d.

The details of BE, TBE calculations are shown in Table S9 of the supplementary material

^a^Reversed CIP priority

*n.d*. the conformation was not detected in the docking studies

In the Prelog complexes, the keto groups of the substrates form hydrogen bond interactions with Tyr^154^ and Ser^141^, while the alkyl or ester side chains extend into a hydrophobic pocket (lined by Tyr^93^, Tyr^151^, Tyr^154^, Leu^197^, and Thr^152^). In this position, the nicotinamide ring is located above the keto group of the substrates, and their aromatic rings point toward the active site entrance. As a result, even very bulky substituents (such as a biphenyl ring in **7**) do not interfere with proper substrate binding. However, it seems that while the hydrophobic pocket can host a substantial substituent, it cannot accommodate phenyl rings as shown by lack of binding poses for benzophenone. Compared to the corresponding Prelog complexes, the modeled anti-Prelog complexes exhibited longer distances between the NADH nicotinamide and the benzylic carbon atoms of the substrates (by 0.26–0.5 Å) and weaker H-bonds with Tyr^154^ (dC = O^…^HO-Tyr^154^ extended from 1.9 to 2.3 Å), in addition to the already mentioned less favorable substrate-binding energetics. Moreover, the alternate substrate conformations of the anti-Prelog models positioned the phenyl rings at about the same region as the Prelog models, but forced the alkyl/ester side chains into an alternative binding pocket, bringing them in close proximity to the NADH nicotinamide ring, which resulted in loosened binding due to steric interferences (as indicated by higher values of vdW interactions with NADH observed for anti-Prelog complexes). The limited space of the alternative binding pocket is also the reason why models of anti-Prelog complexes were only obtained for substrates with methyl groups (**1**, **21**, **22**), but not for those with more bulky, substituted alkyl side chains such as **28**, **29**, or **43**.

The analysis of interaction energies showed that the strong stereospecificity of PEDH, at least for substrates with small side chains, originates mainly from their different electrostatic interactions with Tyr^154^ and vdW interactions with hydrophobic residues of the active site (especially Tyr^94^, Leu^186^, and NADH, see supplementary material Table S4–S6), which favors the reaction in Prelog and impedes it in anti-Prelog complexes.

### Batch reactor tests and modeling of enzyme reactivity

The optimal content of the IPA in the batch reactor has been assayed in three independent reactor test (Fig. S3 of the supplementary material). The results show that the minimum concentration of IPA (14 %) allows conversion of only 25 % of initially added 100 mM of acetophenone. The increase to 60 % IPA dramatically increases the conversion yield (76 % of 200 mM acetophenone). The increase of IPA content to 95 % still improves slightly the final conversion yield (82 % of 200 mM acetophenone) but at the expense of lowering the solubility of some substrates (especially **8** and **9**). As a result, the 60 % IPA content was selected as a good compromise for reactor efficiency and good substrate loading.

**Fig. 3:**
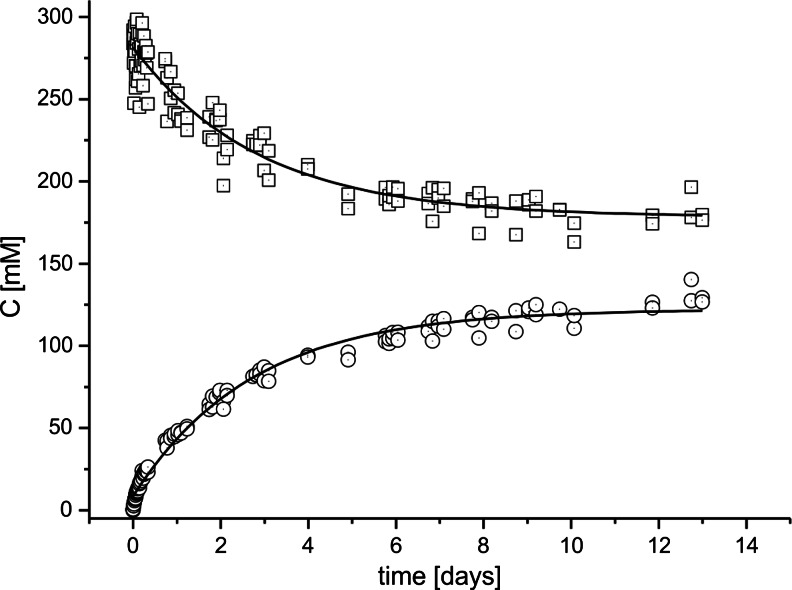
Reaction progress of 4-ethylacetophenone reduction. *Squares* depict concentrations of 4-ethylacetophenone (substrate), *circles* those of 1-(4-ethylphenyl)ethanol (product)

Twenty-two batch reactor tests were performed with nine different acetophenone derivatives (**1**, **4**, **8**, **9**, **10**, **20**, **22**, **33**, **34**) that are characterized by different electron donor/acceptor characteristics of their *para* substituents in the aromatic rings. For the batch reactor series with 50-mM initial substrate concentrations, >80 % conversion was obtained in most of the tested cases with the exception of *p*-OH acetophenone (**22**—conversion yields of 61 and 51 % for 47- and 55-mM batch reactors). Therefore, these experiments did not allow to obtain information about the influence of the substituents on the final conversion yields. However, the batch reaction series with 300-mM initial substrate concentrations yielded mostly partial conversions of the tested compounds in the range of 20 to 100 %, which were apparently limited by the equilibria reached between reduction of the respective ketones and IPA oxidation. Because of the low solubility of substrates **8** and **9** under reaction conditions, they were excluded from the series starting with 300-mM initial concentration. The observed conversion yields are collected in Table 3, while the individual progress curves of the batch reactors are shown in Fig. 3 and in supplementary materials (Fig. S4A–I).

**Table 3:** Parameters of ketone reduction assays in batch reactors

No.	Substrate	Substrate initial concentration (C_0_)	Product final concentration (C^∞^)	Reaction time [h]	Conversion (%)	Equilibrium constant Log K^ex^
1	Acetophenone	47	47	5	100	
		45	45	3	100	
		286	152	88	53	2.24
4	4′-ethylacetophenone	47	46	17	98	
		292	127	180	43	1.92
8	4′-acetylphenyl methanesulfonate	52	41	2	79	
9	4′-nitroacetophenone	47	47	2	100	
		51	51	1	100	
10	4′-fluoroacetophenone	52	51	4	98	
		47	47	3	100	
		107	107	24	100	
		356	273	113	74	2.81
		335	159	30	47	2.15^a^
20	4′-methoxyacetophenone	49	38	16	78	1.75^a^
		55	46	17	84	1.06
		309	62	39	20	1.15
22	4′-hydroxyacetophenone	47	24	15	51	1.43
		55	30	30	60	1.65^a^
		330	80	52	24	1.46
**33**	4′-chloroacetophenone	45	39	3	87	
		273	252	57	92	3.48
**34**	4′-bromoacetophenone	50	49.8	2	100	
		52	5	3	100	
		275	258	63	91	3.41

Initial concentrations of substrates (C_0_), final concentrations of products C^∞^, reaction times at which the 95 % of the final product concentration was reached, and final conversion yields

^a^Values of K^ex^ excluded from further analysis due to lack of points in the equilibrium region or a poor quality of fit to the final points to the rest of progress curve

In the batch reactor system used in this study, PEDH catalyzes two reversible processes: (i) reduction of ketones to alcohols using NADH/H^+^as cosubstrate, yielding the respective alcohol product and NAD^+^ and (ii) oxidation of isopropanol (IPA) with NAD^+^ yielding acetone, NADH, and H^+^. These processes follow the following equations:

1 $$ \mathrm{ketone} + \mathrm{NADH} + {\mathrm{H}}^{+}\ \leftrightarrow \mathrm{alcohol} + {\mathrm{NAD}}^{+} $$

2 $$ \mathrm{I}\mathrm{P}\mathrm{A} + {\mathrm{NAD}}^{+}\ \leftrightarrow \mathrm{acetone} + \mathrm{NADH} + {\mathrm{H}}^{+} $$

During the reaction, the proton concentration is maintained by a phosphate buffer (pH 5.5) while excess of IPA (approximately 10 M) ensures a pseudo-first-order kinetics of NADH recovery. Because of the applied NADH regeneration system, the concentration of NADH and NAD^+^ should remain constant during the turnover reaction, and the overall reaction can be formulated as follows:

3 $$ \mathrm{ketone}+\mathrm{I}\mathrm{P}\mathrm{A}\kern0.24em \underset{{\mathrm{k}}_{\hbox{-} 1}}{\overset{{\mathrm{k}}_1}{\rightleftharpoons }}\kern0.24em \mathrm{alcohol}+\mathrm{aectone} $$

Assuming a (pseudo) first-order reversible reaction kinetics gives the following equilibrium constant:

$$ K=\frac{\left[ alcohol\right]\left[ acetone\right]}{\left[ ketone\right]}=\frac{{\left[ alcohol\right]}^2}{\left[ ketone\right]} $$

We have performed a progress curve analysis of seven batch reactor test from the 300-mM series and two of the 50-mM series that reached final equilibrium, allowing determination of equilibrium constants K. The collected data (see Table 3) show the values of experimental equilibrium constants K^ex^ for six substrates (**1**, **4**, **10**, **20**, **22**, **33**, and **34**). These values (log K^ex^) correlate very well with theoretical log K values for the same substrates derived from thermochemical calculations (*R* ^2^ = 0.98; see Fig. S5 of the supplementary material). Moreover, the K^ex^ values also correlate very well to the respective ∆∆G values of alkoxy anion formation for the different substrates (*R* ^2^ = 0.96). It should be underlined that the actual acetone concentration in the reactors was not monitored. As acetone is the most volatile reagent of the reaction mixture, the frequent probing of reactors during longer reaction runs (up to 8 days) might also have resulted in a decrease of its concentration, possibly resulting in subsequent increase of product content and overestimation of the reported K^ex^ values (Goldberg et al. 2006).

Unfortunately, the rate constants cannot reliably be estimated from our data because of the longtime span of the experiments conducted for the 300-mM series and unsufficient numbers of sampling points during late stages of the experiments.

### Modeling reaction progress in batch reactors

The reaction progress in batch reactor tests was modeled with ANNs that predicted product formation as function of reaction time, based on experimentally collected data and characteristics of the studied substrates (Table 3). Initially, we aimed at development of a single model that could describe the whole range of the concentrations. However, it turned out that the progress curve data sets that started with either 50- or 300-mM substrate concentrations varied too much in time spans and information character. The data inhomogeneity prevented us from developing a single model of satisfactory quality covering the whole range of initial concentrations and reaction times. The obtained model 1 exhibited good prediction capability only for experiments from the 300-mM series, providing reliable information on the final conversion.

In a second approach, we limited the time span of all experiments that were fed into the ANN model to that observed for the 50-mM series (i.e., 1000 min). Thereby, we could use data from both the 50- and 300-mM series for an equal, shorter time span. This modification of the data set enabled the development of a much more robust prediction model (model 2) which predicted correctly the initial parts of the progress curves.

**Fig. 4:**
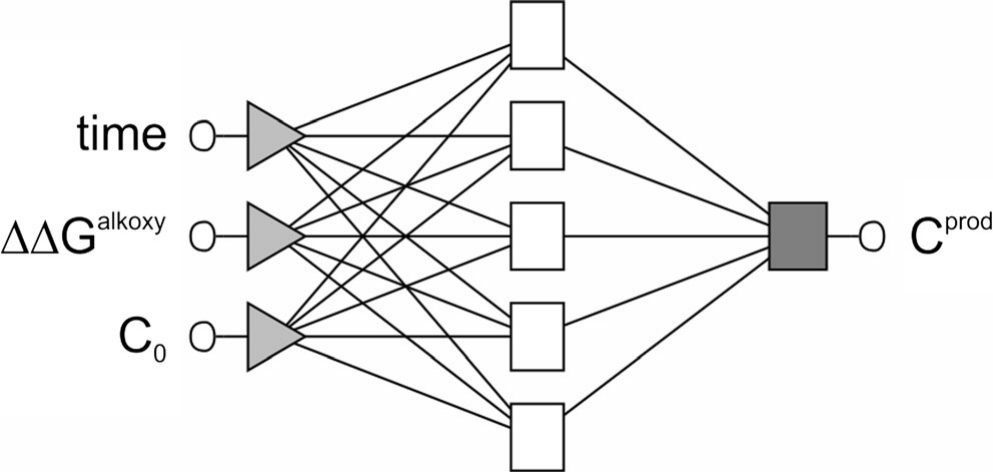
Schematic representation of the MLP 3-5-1 neural network. *Triangles* input neurons, *white rectangles* hidden neurons, *gray rectangle* output neuron, *circle* input and output numerical variables

The optimal architecture of both models comprised three input neurons, five hidden neurons, and one output neuron (MLP 3-5-1; Fig. 4). As input variables, C_0_, ΔΔG^alkoxy^ values, and reaction time (t) were used. Optimization of the number of hidden neurons (2–10) of the internal layer showed that the error of the validation initially decreases with more hidden neurons, but stays about constant with for 5 or more.

Both models were trained with 100 epochs of a back-propagation algorithm followed by a conjunct gradient algorithm with inertia (175 and 260 epochs for models 1 and 2, respectively). The values of weights (constants of linear aggregation functions) as well as the types of activation and aggregation functions used for both neural models are shown in the supplementary material (Tables S7–8).

**Fig. 5:**
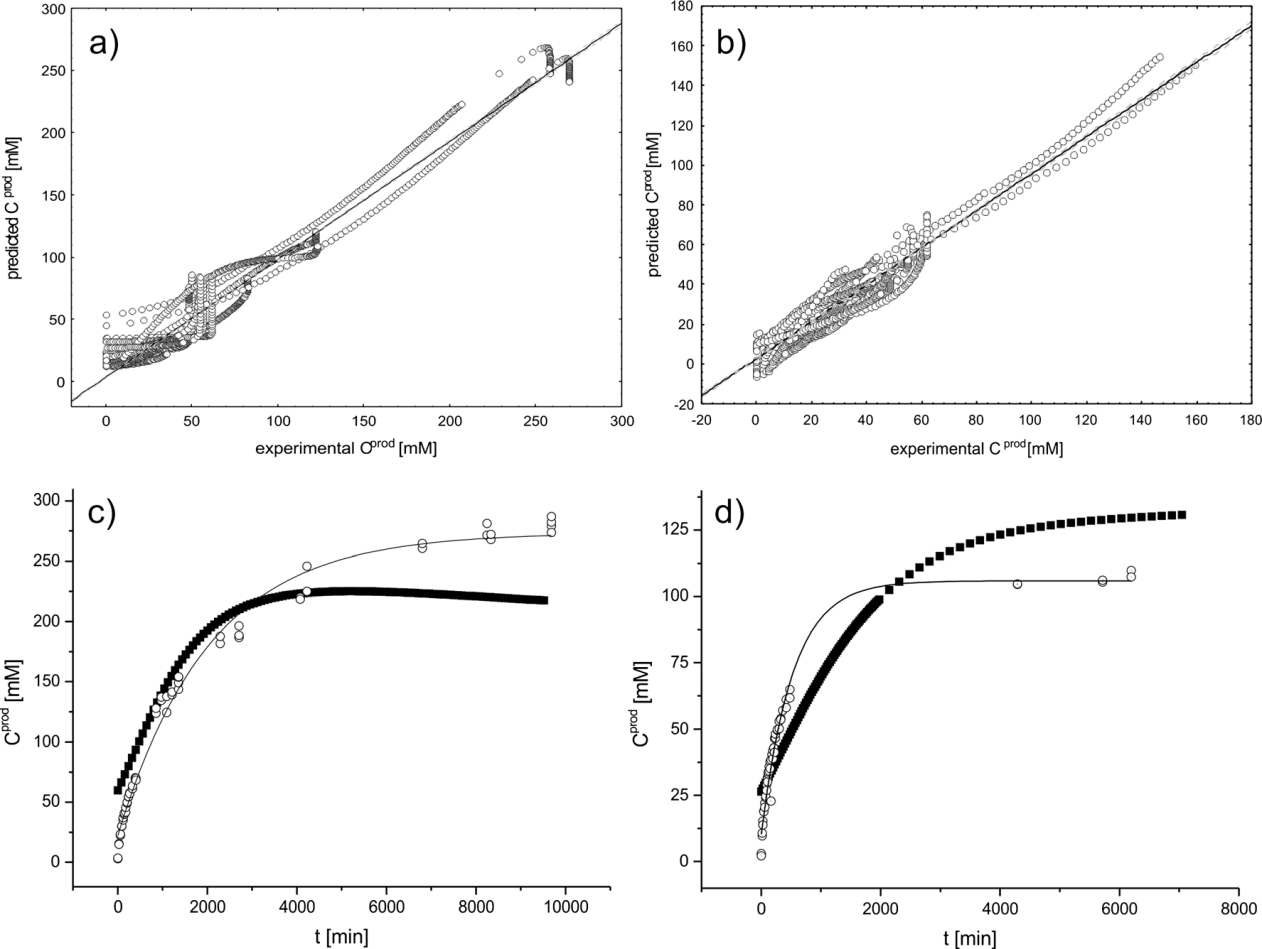
Prediction capabilities of the neural network models: **a** scatter plots of all experimental versus all predicted product concentrations obtained for model 1 over the whole reaction time span (*R* ^2^ 0.96) and **b** scatter plots for model 2 over 1000-min reaction time (*R* ^2^ = 0.94), **c** prediction of progress curves (concentrations of 1-(4-fluorophenyl)ethanol ) in external validation set (4′-fluoroacetophenone) during whole reaction time span-model 1 C_0_ 356 mM (*R* ^2^ = 0.76), **d** the same for model 1 C_0_ 107 mM (*R* ^2^ = 0.74); *circles* experimental data points, *line* mono-exponential fit to the experimental points, *black squares* neural network predictions

Model 1 was characterized by very good prediction capabilities (*R* ^2^ of 0.97, 0.97, and 0.98, respectively, for learning, validation, and test groups, Fig. 5a) and similar errors for learning, validation, and test subsets (0.0354, 0.0358, 0.0368, respectively). However, model 1 exhibited markedly low prediction accuracy in the range of 0–1000 min (*R* ^2^ of 0.798).

Model 2 predicted changes in product concentration in the early phases (up to 1000 min) with excellent confidentiality values (*R* ^2^ of 0.94, 0.93, and 0.93, respectively, for learning, validation, and test groups, Fig. 5b) and showed errors for learning, validation, and test on the same level and slightly lower than those of model 1 (0.0321, 0.0327, 0.0337, respectively).

The generalization capabilities of both models were tested on an external validation set, i.e., four experiments conducted with 4′-fluoroacetophenone (10). Model 2 exhibited moderate generalization capabilities and predicted the concentration of the alcohol product 10 with *R* ^2^ of 0.962, 0.359, and 0.757 for experiments started with 50, 100, and 300 mM of ketone substrate. In contrast, model 1 exhibited much worse predictive performance for the 50-mM external validation set with *R* ^2^ 0.496 but better *R* ^2^ for 100 and two experiments of 300 mM for an external validation set (0.738, 0.766, and 0.869, respectively) (see Fig. 5c, d).

**Fig. 6:**
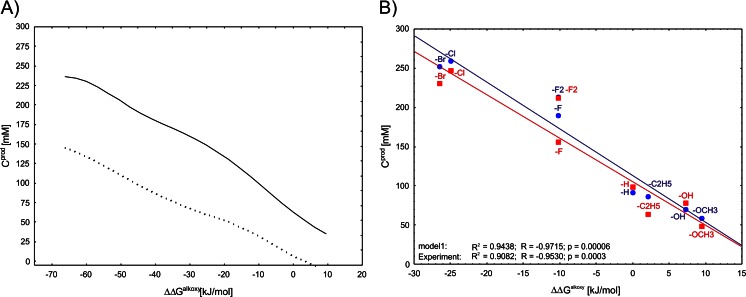
**a** Response curves for model 1 (*solid line*) and model 2 (*dots*) showing dependence of predicted product concentrations from ΔΔG^alkoxy^. The response curves are generated as a 2D projection of a model behavior assuming all the other input variables are constant at their average values for a given dataset. **b** Correlation scatter plot of ΔΔG^alkoxy^ versus product concentrations after 48-h reaction time for reactors of 300-mM series: *red squares* experimental values, *blue circles* model 1 predictions. In both cases, high linear correlations are observed between ΔΔG^alkoxy^ and concentrations of products (*R* ^2^ > 0.90)

Another aim of the modeling of progress curves with ANN was to figure out which factors influence enzyme reactivity, although multidimensional ANN models are generally very difficult to study analytically. A frequently used approach to determine the relationships of nonlinear data is response curve analysis, which provides graphical relations between individual independent variables and the output variable (product concentration), assuming average values for the other input variables. As long as the input variables used in the model are orthogonal (not interrelated), such analysis delivers useful information on the modeled phenomenon under studies. The most interesting relationship predicted from our models is the correlation of strong electron-withdrawing characters of the *para* substituents with higher product concentrations obtained (see Fig. 6a, the other response curves are available in Fig. S6 of the supplementary material). Similar relation can be observed by plotting product concentrations observed after 48 h in reactors of 300-mM series versus ΔΔG^alkoxy^ parameter. The linear relationship is observed both for experimental values (Fig. 6b circles) and values predicted by model 1 (Fig. 6b squares).

It appears that the ΔΔG^alkoxy^ parameter is interchangeable to Hammett σ_p_ or calculated log K (as expected based on correlation analysis; see Fig. S1) and neural models of similar quality and architecture (MLP 3-5-1) can be achieved with these descriptors (*R* ^2^ of 0.98 for neural model utilizing σ_p_ and average *R* ^2^ of 0.97 for model utilizing log K). Therefore, it must be underlined that the observed correlation effect (Fig. 6) describes combination of collinear kinetic and thermodynamic factors that influence productivity of the reaction system.

## Discussion

**Fig. 7:**
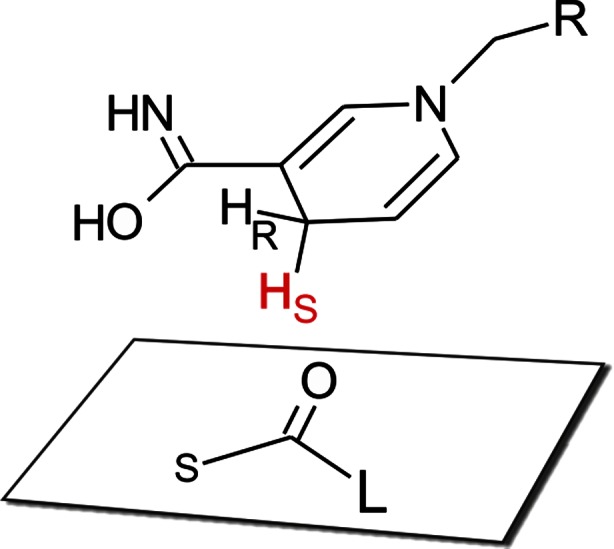
Pro-(*S*) hydride of NADH delivered to the *Re* face of ketone. S and L denote small and large ligands, respectively

PEDH shows a broad substrate specificity in enantiospecifically reducing more than 50 carbonyl compounds including ketones and β-keto esters with different lengths and substitutions of the alkyl chain and with aromatic or heterocyclic rings. Almost all investigated substrates were reduced to a single enantiomer of the corresponding alcohol following Prelog’s rule (Rodrigues et al. 2004), the only exceptions being products chemically racemized at acidic pH after conversion (substrates **21** and **22**). Hydride delivery from the reduced nicotinamide cofactor occurs to the *Re* face of the carbonyl group when the large group L (aromatic ring) and the small group S (alkyl side chain) substitute the carbonyl as shown in Fig. 7. Our modeling studies and thermodynamic calculations confirmed that this binding mode is the only feasible one in PEDH. Theoretical docking of substrates in anti-Prelog orientations in a catalytically feasible manner (as judged by formation of H-bonds with Tyr^154^ and Ser^141^) was only possible for substrates with relatively small (methyl) side chains and led to complexes with much less favorable system energies (approximately 21–33 kJ/mol difference to the respective Prelog complexes). Exposition of the *Re* face of the substrate toward NADH results in the formation of the (*S*)-enantiomer of the alcohols for all of the studied compounds except for those with a reversed CIP priority. PEDH specifically distinguishes between large and small substituents of the ketones according to the Prelog classification and does not convert ketones with two large substituents such as benzophenone. The enzyme is obviously unable to accommodate the additional phenyl ring of benzophenone inside its hydrophobic binding pocket as suggested by previous modeling studies (Hӧffken et al. 2006) and confirmed by docking studies.

The mechanism of ADHs is further classified according to which H atom is transferred from NADH. In PEDH, the NADH cofactor is bound in the *syn* conformation, which exposes the pro-(*S*) hydrogen atom toward the enzyme active site as reactant for ketone reduction (Nambiar et al. 1983). According to Benner et al. (Nambiar et al. 1983), the H_S_ hydrogen atom has a stronger reduction potential than the H_R_, making *syn*-reactive ADHs the better choice for reduction of ketones, although the reaction equilibrium still favors the keto form.

We have shown that PEDH is an excellent potential catalyst for industrial application as it catalyzes the recovery of NADH with IPA added to the reaction system as a sacrificial cosubstrate. PEDH shows an extremely high tolerance to IPA in comparison to other ADHs (Itoh et al. 2002; Kosjek et al. 2004). Due to this high tolerance, we could perform reactions in high concentrations of IPA (60 %) that additionally increased the solubility of water-insoluble substrates in the batch reaction system up to 300 mM. As the reaction proceeded even in 95 % IPA as solvent (see supplementary material, Fig. S3), even higher reactor loading is achievable. IPA as cosubstrate is oxidized to acetone in a NAD^+^-coupled reaction, thus conveniently recovering the NADH required for ketone reduction. The high applicable concentrations of IPA (60 % equals to 10 M) compared to the ketone substrates helps in driving the equilibrium toward efficient ketone reduction, even if IPA is only a poor substrate for PEDH (Hӧffken et al. 2006).

Our studies proved the applicability of a whole-cell reaction system of *E. coli* cells with recombinant PEDH for preparative alcohol syntheses. Increasing the substrate concentrations from 50 mM (100 % conversion for all tested *p*-substrates except *p*-OH-acetophenone) to 300 mM led to a decrease of the final conversions (approximately to 50 %), even if the total product concentrations were still higher for most of the tested ketones. The conversions apparently depend on the relations between equilibrium constants of ketone reduction and IPA oxidation. However, most substrates were still satisfactorily converted under the conditions tested, and knowing the parameters of the desired substrate, appropriate substrate concentrations to archive complete conversion can easily be applied. Finally, products of high purity may be obtained by simple extraction procedures.

The analysis of initial slopes of the progress curves shows that the acetophenone derivatives are converted to alcohols at different rates. It was previously reported that the electron density at the carbonyl group influences the rate of the hydride transfer reactions (Naik et al. 2012; Zhu et al. 2006a, b). Unfortunately, the enzyme kinetic constants determined for isolated PEDH cannot be directly applied to describe the behavior of the reactor system. This is due to the fact that the enzyme in the batch reactor system is conducting reactions in both directions in a complex IPA/acetone/water solvent medium, with unknown intracellular NADH/NAD levels, reaching either full substrate conversion or a final equilibrium between substrate reduction and IPA oxidation. Further factors precluding the estimation of rate constants from analysis of the product formation progress curves are complex phase transitions, partitioning effects between the reaction medium and bacterial cells, potential concentration changes of the rather volatile acetone during long-lasting experiments, and the eventual decrease of enzyme activity during the reactor runs. However, we were able to estimate experimental equilibrium constants for six compounds and correlate these with theoretically obtained values (yielding high linear correlations to either calculated K or ∆∆G^alkoxy^).

Meanwhile, the neural networks can be used for description of progress curves of the whole-cell reactors. We provide models capable of predicting product formation with substrate concentrations in the 50–300 mM range. Our findings confirm the observation that acetophenone derivatives containing an electron-withdrawing group at the *para* position were reduced faster (and more completely) than those with an electron-donating group. For *para*-substituted acetophenone derivatives, the electron donor-acceptor effect of a given substituent is described by ∆∆G^alkoxy^ values which can replace the Hammett σ_p_ constant (Uwai et al. 2008) as descriptor for reactivity in ketone reduction especially for compounds with unknown Hammett parameters. Similar observations can be reached by plotting ∆∆G^alkoxy^ versus experimental product concentrations at the selected (a prori) reaction time for reactor series where equilibrium effect was observed (i.e., 300 mM). A strong linear relationship (*R* ^2^ = 0.94, Fig. 6B) indicates that the more electron-withdrawing group is introduced into the substrate, the more thermodynamic equilibrium is shifted further into alcohol product side. Interestingly, introduction of the electron-withdrawing groups seems to be a common cause of both acceleration of the reaction rate (decrease of transition state barrier as modeled by ∆∆G^alkoxy^) and the shift in the equilibrium constant (as modeled by log K). Finally, the neural models exhibited relatively good generalization capabilities (with just a slight overestimation of the product concentration) as they were able to predict reaction progress of reduction of 4′-fluoroacetophenone which was not used for model development.

A whole-cell reaction system with recombinant PEDH was employed for enantioselective reduction of ketones, using IPA as solvent and sacrificial substrate for NADH recovery. It was established as a convenient method for synthesizing a wide range of chiral alcohols. The high enantiospecificity of the enzyme originates from the structure of the ternary ES complex. Electron-withdrawing substituents in the ketone substrates enhance the reaction rate and thermodynamically favor alcohol formation, resulting in both faster conversion and shifting of the equilibrium toward the product. A simple ANN model has been trained based on the experimental data of ketone reduction reactors and used as an efficient prediction tool for assessing their performance.

## Electronic supplementary material

Below is the link to the electronic supplementary material.

Supplementary material The supplementary material contain descriptors for*para*-substituted substrates used in ANN modeling,details of LC/MS analysis, details of binding energy calculations, binding modes of **21** and **22**, scatter plots showing linear correlations between Hammett σ_p_ and calculated descriptors: ∆∆G^alkoxy^ and log K, response curves for model 1 and model 2, progress curves obtained for conversion of acetophenone with various concentrations of IPA, information about generation of points for ANN, experimental reaction progress curves, correlation plot of predicted vs. experimental log K and a list of chemicals. (PDF 1448 kb)
