# Supplementary material for: Asymmetric reduction of ketones and β-keto esters by (S)-1-phenylethanol dehydrogenase from denitrifying bacterium Aromatoleum aromaticum
Source: Appl Microbiol Biotechnol. 2014 Dec 31;99(12):5055–69. doi: 10.1007/s00253-014-6309-z (PMC4445480; doi:10.1007/s00253-014-6309-z)
Supplement: Supplementary file 1 — The supplementary material contain descriptors forpara-substituted substrates used in ANN modeling,details of LC/MS analysis, details of binding energy calculations, binding modes of 21 and 22, scatter plots showing linear correlations between Hammett σp and calculated descriptors: ∆∆Galkoxy and log K, response curves for model 1 and model 2, progress curves obtained for conversion of acetophenone with various concentrations of IPA, information about generation of points for ANN, experimental reaction progress curves, correlation plot of predicted vs. experimental log K and a list of chemicals. (PDF 1448 kb) [file 253_2014_6309_MOESM1_ESM.pdf]

Applied Microbiology and Biotechnology

Supplementary Information

Asymmetric reduction of ketones and β-keto esters by (*S*)-1-phenylethanol dehydrogenase from denitrifying bacterium *Aromatoleum aromaticum*

A. Dudzik[a], W. Snoch[a,b], P. Borowiecki[c], J. Opalinska-Piskorz[a], M. Witko[a], J. Heider[d], M. Szaleniec[a*]

[a] Jerzy Haber Institute of Catalysis and Surface Chemistry Polish Academy of Sciences, Niezapominajek 8, 30-239 Kraków, Poland *Maciej Szaleniec: Fax: (+48)124251923 E-mail: ncszalen@cyfronet.pl
[b] Cracow University of Technology, Faculty of Chemical Engineering and Technology, Department of Biotechnology and Physical Chemistry, Warszawska 24 St. 31-155 Krakow, Poland
[c] Warsaw University of Technolology, Faculty of Chemistry, Noakowskiego 3, 00-664 Warsaw, Poland.
[d] Laboratory for Microbial Biochemistry, Philipps University of Marburg, Karl-von-Frisch Strasse 8, D-35043 Marburg, Germany

Table S1 HPLC/MS data of alcohol products including their normal phase APCI mass signals, retention times based on UV-Vis detection (RT/UV-Vis) and IPA/n-hexane mobile phase composition

No	product	+ NP-APCI	RT/UV-vis	IPA/n-hexane
**aromatic ketones**				
1	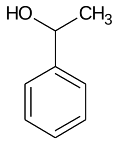 1-phenylethanol	n.d.	13.81	10/90
2	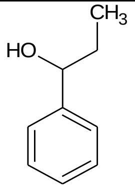 1-phenylpropan-1-ol	$[M-OH]^+$ 119	12.73	10/90
3	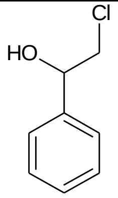 2-chloro-1-phenylethanol	n.d.	12.66	15/85

4	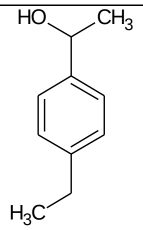 1-(4-ethylphenyl)ethanol	$[M\text{-}OH]^+$ 133	9.91	10/90
5	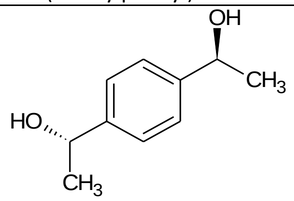 1-(4-(1-hydroxy-ethyl)-phenyl)-ethanol	$[M\text{-}OH]^+$ 133<br>$[M\text{-}H_2O\text{-}OH]^+$ 131	22.33	10/90
6	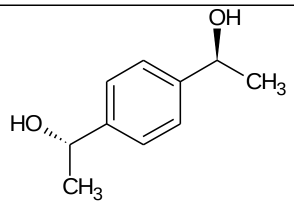 1-(4-(1-hydroxy-ethyl)-phenyl)-ethanol	$[M\text{-}OH]^+$ 133<br>$[M\text{-}H_2O\text{-}OH]^+$ 131	22.33	10/90
7	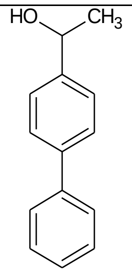 1-(biphenyl-4-yl)ethanol	$[M\text{-}OH]^+$181	17.64	10/90
8	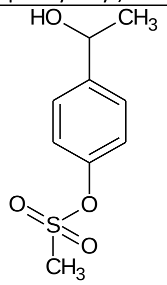 4-(1-hydroxyethyl)phenyl methanesulfonate	$[M\text{-}OH]^+$ 199	25.25	30/70

9	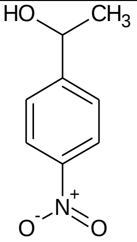<br>1-(4-nitrophenyl)ethanol	[M] + 168	13.00	20/80
10	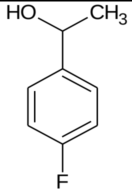<br>1-(4-fluorophenyl)ethanol	[M-OH]+ 123	11.37	10/90
11	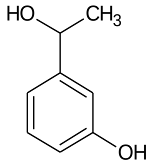<br>3-[(1)-1-hydroxyethyl]phenol	[M-OH]+ 121	18.00	10/90
12	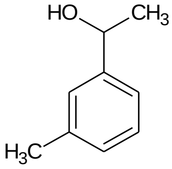<br>1-(3-methylphenyl)ethanol	[M-OH]+ 106	10.97	10/90
13	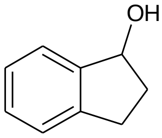<br>1-indanol	[M-OH]+ SIM 117	11.79	20/80
14	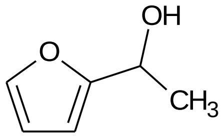<br>1-(furan-2-yl)ethanol	[M-OH]+ 95	17.02	10/90
15	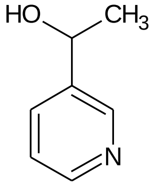<br>1-(pyridin-3-yl)ethanol	[M]+ 124	12.34	15/85

16	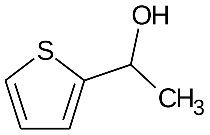 1-(thiophen-2-yl)ethanol	$[M-OH]^+$ 111	17.25	10/90
17	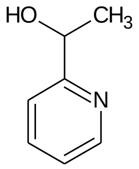 1-(pyridin-2-yl)ethanol	$[M-OH]^+$ 106	10.86	10/90
18	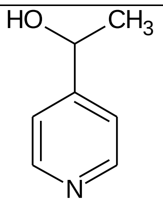 1-(pyridin-4-yl)ethanol	$[M]^+$ 124	13.60	15/85
19	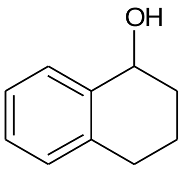 1,2,3,4-tetrahydronaphthalen-1-ol	$[M-OH]^+$	12.40	15/85
20	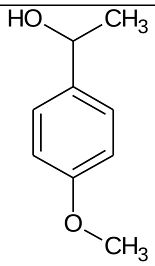 1-(4-methoxyphenyl)ethanol	$[M-OH]^+$ 135	14.39	25/75
21	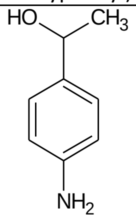 1-(4-aminophenyl)ethanol	118	23.64<br>26.64	30/70
22	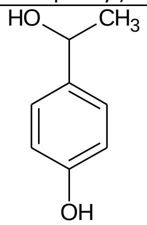 4-(1-hydroxyethyl)phenol	$[M-OH]^+$ 121	24.04 27.24	10/90

23	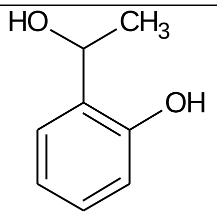<br>2-(1-hydroxyethyl)phenol	[M-OH]+ 121	14.49	10/90
24	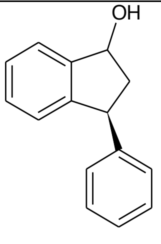<br>(S)-3-phenyl-1-indanol	[M-OH]+<br>147	12.72	10/90
25	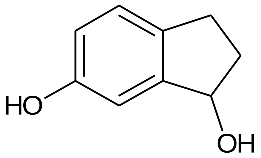<br>2,3-dihydro-1H-indene-1,6-diol	[M-OH]+ 133	18.52	10/90
26	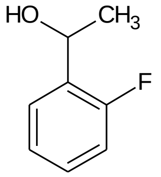<br>1-(2-fluorophenyl)ethanol	[M-OH]+ 123	10.53	10/90
27	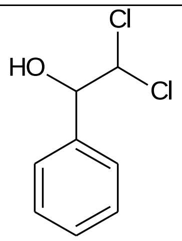<br>2,2-dichloro-1-phenylethanol	n.d.	15.06	10/90
28	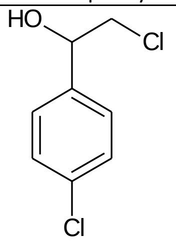<br>2-chloro-1-(4-chlorophenyl)ethanol	n.d.	15.10	10/90
29	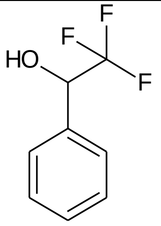<br>1-phenyl-2,2,2-trifluoroethan-1-ol	n.d.	11.27	10/90

30	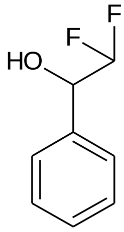 1-phenyl-2,2-trifluoroethan-1-ol	n.d.	16.75	10/90
31	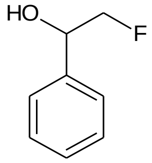 2-fluoro-1-phenylethan-1-ol	n.d.	16.00	10/90
32	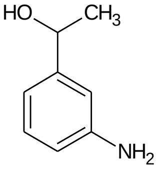 1-(3-aminophenyl)ethanol	n.d.	15.97	35/65
33	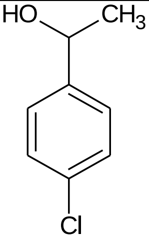 1-(4-chlorophenyl)ethanol	n.d.	9.38	15/85
34	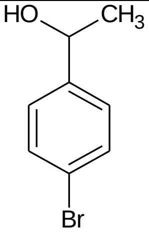 1-(4-bromophenyl)ethanol	n.d.	9.62	15/85
35	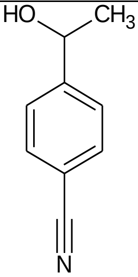 4-(1-hydroxyethyl)benzonitrile	$[M]^+$ 148	15.13	15/85
36	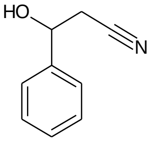 3-hydroxy-3-phenylpropanenitrile	$[M]^+$ 148	17.78	20/80

37	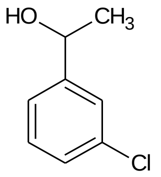<br>1-(3-chlorophenyl)ethanol	n.d.	9.96	15/85
38	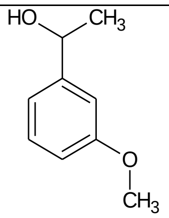<br>1-(3-methoxyphenyl)ethanol	n.d	16.81	15/85
39	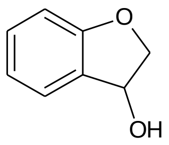<br>2,3-dihydro-1-benzofuran-3-ol	n.d.	10.51	15/85
40	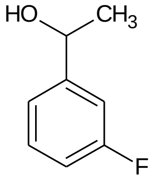<br>1-(3-fluorophenyl)ethanol	$[M-OH]^+$ 123	11.20	10/90
41	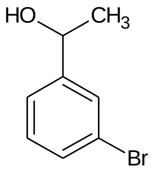<br>1-(3-bromophenyl)ethanol	n.d.	10.76	15/85
42	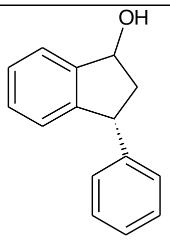<br>(*R*)-3-phenyl-1-indanol	$[M-OH]^+$ 147	14.23	10/90
**β-keto esters**				

43	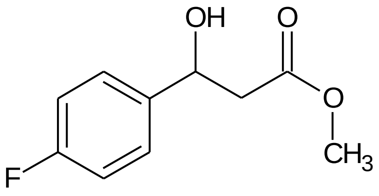 methyl 3-(4-fluorophenyl)-3-hydroxypropanoate	n.d.	13.38	25/75
44	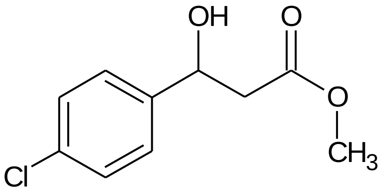 methyl 3-(4-chlorophenyl)-3-hydroxypropanoate	n.d.	13.45	25/75
45	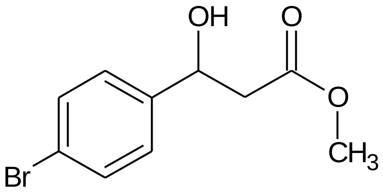 methyl 3-(4-bromophenyl)-3-hydroxypropanoate	n.d.	14.21	25/75
46	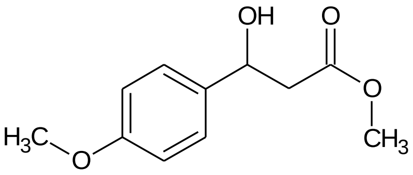 methyl 3-hydroxy-3-(4-methoxyphenyl)propanoate	n.d.	21.60	30/70
47	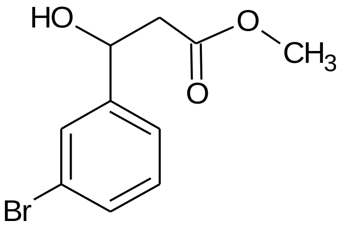 methyl 3-(3-bromophenyl)-3-hydroxypropanoate	n.d.	19.3	25/75
48	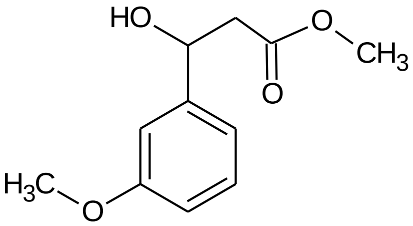 methyl 3-hydroxy-3-(3-methoxyphenyl)propanoate	n.d.	31.64	30/70
49	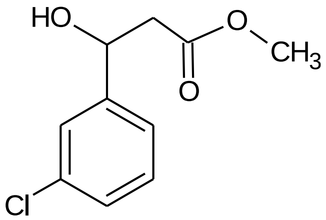 methyl 3-(3-chlorophenyl)-3-hydroxypropanoate	n.d.	16.75	25/75

50	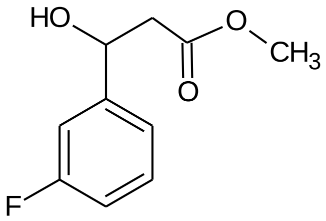 methyl 3-(3-fluorophenyl)-3-hydroxypropanoate	n.d.	12.44	25/75
51	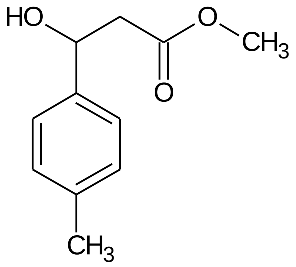 methyl 3-hydroxy-3-(4-methylphenyl)propanoate	n.d.	12.05	30/70
52	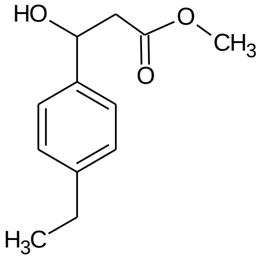 methyl 3-(4-ethylphenyl)-3-hydroxypropanoate	n.d.	11.86	20/80
53	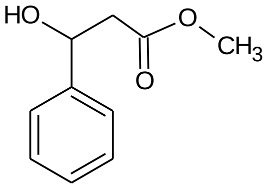 methyl 3-hydroxy-3-phenylpropanoate	n.d.	15.47	20/80

Table S2 List of compounds

**Chemical name and purity**	**Supplier**
1-phenylethanol, 98%	Aldrich
(S)-1-phenylethanol, 98.5%	Fluka
(R)-1-phenylethanol, 97%	Aldrich
1-phenylpropan-1-ol, 97%	Aldrich
(S)-1-phenylpropan-1-ol, 99%	Aldrich
1-phenylprop-2-en-1-ol 99%	Aldrich
(R)-1-phenylprop-2-en-1-ol, 95%	Aldrich
1-phenylprop-2-yn-1-ol, 98%	Alfa Aesar
1-(4-methylphenyl)ethanol, ≥97.0%	Aldrich
1-(4-fluorophenyl)ethanol, 97%	Alfa Aesar
(S)-1-(4-fluorophenyl)ethanol , 97%	Aldrich
1-(4-methoxyphenyl)ethanol, 95%	Alfa Aesar
1-(2-methylphenyl)ethanol, 98%	Alfa Aesar
2,3-dihydro-1*H*-inden-1-ol, 98%	Aldrich
(S)-2,3-dihydro-1*H*-inden-1-ol, 99%	Fluka
1,2,3,4-tetrahydronaphthalen-1-ol, 97%	Aldrich
(S)-1,2,3,4-tetrahydronaphthalen-1-ol ,99%	Aldrich
(S)-1,2,3,4-tetrahydronaphthalen-1-ol , ≥99.0%	Fluka
1-(furan-2-yl)etanol, ≥99.0%	Aldrich
(S)-1-(furan-2-yl)ethanol, 94%	Fluka
(S)-1-(thiophen-2-yl)ethanol, ≥99.0%	Aldrich
(R)-1-(thiophen-2-yl)ethanol, ≥98.5%	Fluka
1-([1,1'-biphenyl]-4-yl)ethanol, 97%	Aldrich

Table S3 Descriptors for *para*-substituted substrates used in ANN modeling. Strong electron withdrawing effects of the substituent (positive σ_p) are proportional to more negative ΔΔG values of alkoxy anion formation (reference ΔG calculated for acetophenone -264.4 kJ/mol) – see Fig. S1 of the Supplementary Information.

-R	σ_p	$\Delta\Delta G^{alkoxy}$ [kJ/mol]	Log K	Log P	MR
-OH	-0.37	7.32	-3.745	1.35	38.155
-OCH3	-0.27	9.49	-3.824	9.88	42.924
-C2H5	-0.15	2.15	-3.045	2.507	46.103
-H	0.00	0	-2.620	1.58	36.461
-F	0.06	-10.2	-2.066	1.72	36.677
-Br	0.23	-26.44	-1.638	2.43	44.084
-Cl	0.23	-24.95	-1.658	2.32	41.266
$-SO_3CH_3$	0.33	-22.6	-2.464	1.217	45.81
$-NO_2$	0.78	-66.19	-0.592	1.53	43.786

Table S4 Calculated interaction energies of acetophenone in PEDH active site in Prelog and anti-Prelog positions.

**Residue**	**Interaction Energy (kJ/mol)**	**VDW Interaction Energy (kJ/mol)**	**Electrostatic Interaction Energy (kJ/mol)**	**Interaction Energy (kJ/mol)**	**VDW Interaction Energy (kJ/mol)**	**Electrostatic Interaction Energy (kJ/mol)**	**Δ IE (AntiP-P) (kJ/mol)**
	**Prelog (pro(*S*))**			**Anti-Prelog (pro(*R*))**			
B_GLY14	0.000	0.000	0.000	0.000	0.000	0.000	0.000
B_GLY18	0.042	-0.004	0.042	0.000	0.000	0.000	-0.042
B_ILE19	-0.100	-0.176	0.075	-0.192	-0.071	-0.121	-0.088
B_GLY20	-0.042	0.000	-0.042	0.000	0.000	0.000	0.042
B_ASN88	0.000	0.000	0.000	0.000	0.000	0.000	0.000
B_ASN89	-0.046	-0.013	-0.029	0.828	0.000	0.828	0.874
B_ALA90	-0.142	-0.004	-0.138	-0.004	0.000	-0.004	0.138
B_GLY91	0.000	-0.050	0.050	0.084	-0.008	0.092	0.084
B_ILE92	-0.063	-0.201	0.138	-0.004	-0.071	0.067	0.059
B_TYR93	-8.745	-8.586	-0.159	-1.920	-1.879	-0.042	6.828
B_PRO94	-0.088	-0.121	0.038	-0.105	-0.109	0.000	-0.021
B_LEU95	-0.075	-0.188	0.113	-0.523	-0.276	-0.247	-0.448
B_ILE96	-0.042	0.000	-0.042	0.071	-0.013	0.084	0.117
B_THR109	-0.021	0.000	-0.021	-0.038	0.000	-0.038	-0.017
B_PHE110	0.000	0.000	0.000	0.000	0.000	0.000	0.000
B_ILE112	0.000	0.000	0.000	0.000	0.000	0.000	0.000
B_ASN113	0.071	-0.025	0.096	-0.146	-0.008	-0.138	-0.218
B_VAL114	0.000	0.000	0.000	0.000	0.000	0.000	0.000
B_SER116	0.000	0.000	0.000	0.000	0.000	0.000	0.000
B_ASN138	0.046	0.000	0.046	-0.013	0.000	-0.013	-0.059
B_LEU139	0.075	-0.054	0.130	-0.067	-0.033	-0.038	-0.146
B_THR140	0.004	-0.293	0.297	-0.226	-0.251	0.025	-0.230
B_SER141	-10.142	-4.833	-5.314	-9.406	-5.556	-3.849	0.736
B_THR142	-5.619	-5.017	-0.598	-4.058	-3.992	-0.071	1.556
B_THR143	-10.510	-9.728	-0.782	-10.309	-10.381	0.071	0.201
B_TYR144	-0.180	-0.205	0.029	-0.326	-0.209	-0.117	-0.146
B_TRP145	-0.460	-0.163	-0.297	-0.418	-0.142	-0.276	0.042
B_LEU146	-4.975	-5.029	0.054	-2.021	-2.109	0.088	2.950
B_ILE148	-5.335	-5.485	0.146	-5.870	-5.870	0.000	-0.536
B_GLU149	-0.268	-0.071	-0.197	-0.715	-0.251	-0.464	-0.444
B_ALA150	-0.117	-0.033	-0.084	-0.100	-0.126	0.025	0.017
B_TYR151	-4.125	-3.971	-0.155	-5.561	-5.402	-0.159	-1.435

B_THR152	0.142	-0.004	0.146	0.347	-0.021	0.368	0.205
B_HIS153	0.000	-0.013	0.013	-0.377	-0.025	-0.351	-0.377
B_TYR154	-12.489	-6.046	-6.448	-11.870	-7.272	-4.598	0.623
B_ILE155	-0.134	-0.301	0.163	-0.607	-0.678	0.071	-0.469
B_SER156	0.155	0.000	0.155	0.084	-0.004	0.088	-0.067
B_THR157	-0.226	-0.013	-0.213	-0.084	-0.008	-0.075	0.142
B_LYS158	-1.397	-0.502	-0.895	-1.042	-0.418	-0.623	0.356
B_ALA159	0.180	0.000	0.180	0.126	0.000	0.130	-0.054
B_ALA160	0.000	0.000	0.000	0.000	0.000	0.000	0.000
B_ASN161	-0.435	-0.008	-0.431	0.033	-0.004	0.038	0.469
B_ILE162	-0.059	0.000	-0.054	-0.025	0.000	-0.025	0.029
B_ALA181	0.000	0.000	0.000	0.000	0.000	0.000	0.000
B_ILE182	-0.243	-0.021	-0.226	-0.155	-0.025	-0.130	0.088
B_ALA183	-0.381	-0.184	-0.197	-0.310	-0.205	-0.109	0.067
B_PRO184	-1.745	-1.824	0.079	-2.678	-2.427	-0.255	-0.937
B_SER185	-5.879	-5.573	-0.310	-4.000	-4.151	0.151	1.879
B_LEU186	-17.209	-17.154	-0.054	-12.824	-12.820	-0.004	4.385
B_VAL187	-1.331	-1.284	-0.046	-0.862	-0.736	-0.126	0.473
B_THR189	-0.285	-0.184	-0.105	-0.067	-0.038	-0.025	0.222
B_THR191	-0.485	-0.180	-0.305	-0.176	-0.033	-0.142	0.310
B_THR192	-2.326	-2.556	0.234	-0.552	-0.569	0.017	1.774
B_SER195	0.377	-0.251	0.628	-0.201	-0.188	-0.013	-0.577
B_LEU197	-11.029	-11.205	0.176	-9.146	-9.000	-0.146	1.883
B_PHE201	-0.544	-0.381	-0.163	-0.594	-0.331	-0.264	-0.050
B_LEU204	-3.715	-3.820	0.105	-1.858	-2.008	0.151	1.858
B_GLN215	-0.033	-0.167	0.130	-0.452	-0.121	-0.331	-0.414
B_LEU220	-0.008	-0.029	0.021	-0.021	-0.025	0.004	-0.013
B_THR239	0.004	0.000	0.004	0.000	0.000	0.000	0.000
B_ALA241	0.172	-0.008	0.180	-0.004	-0.013	0.008	-0.176
B_VAL242	0.000	-0.029	0.025	0.201	-0.033	0.234	0.205
B_ASP243	-0.100	-0.197	0.096	-1.029	-0.159	-0.870	-0.929
B_MET246	-1.435	-1.251	-0.184	-0.527	-0.707	0.180	0.908
B_VAL247	0.105	-0.096	0.201	-0.121	-0.084	-0.038	-0.226
B_NAD256	-16.774	-16.008	-0.761	-5.736	-6.402	0.665	11.033
SUM	-127.997	-113.537	-14.460	-95.567	-85.257	-10.309	

Table S5 Calculated interaction energies of 4′-hydroxyacetophenone in PEDH active site in Prelog and anti-Prelog positions.

Residue	Interaction Energy (kJ/mol)	VDW Interaction Energy (kJ/mol)	Electrostatic Interaction Energy (kJ/mol)	Interaction Energy (kJ/mol)	VDW Interaction Energy (kJ/mol)	Electrostatic Interaction Energy (kJ/mol)	Δ IE (AntiP-P) (kJ/mol)
	**Prelog (pro(*S*))**			**Anti-Prelog (pro(*R*))**			
B_GLY14	0.000	0.000	0.000	0.000	0.000	0.000	0.000
B_GLY18	0.025	0.000	0.025	0.000	0.000	0.000	-0.042
B_ILE19	-0.092	-0.105	0.013	-0.158	-0.067	-0.091	-0.059
B_GLY20	0.017	0.000	0.017	0.000	0.000	0.000	0.038
B_ASN88	0.000	0.000	0.000	0.000	0.000	0.000	0.000
B_ASN89	0.356	-0.008	0.364	0.640	-0.001	0.641	0.841
B_ALA90	-0.092	0.000	-0.092	0.000	0.000	0.000	0.138
B_GLY91	0.033	-0.050	0.079	0.023	-0.007	0.030	0.088
B_ILE92	-0.088	-0.234	0.146	0.008	-0.062	0.071	0.067
B_TYR93	-8.531	-8.058	-0.469	-1.831	-1.584	-0.247	6.820
B_PRO94	-0.172	-0.155	-0.013	-0.177	-0.085	-0.092	0.029
B_LEU95	-0.021	-0.238	0.213	-0.111	-0.204	0.094	-0.410
B_ILE96	-0.038	-0.004	-0.038	-0.124	-0.007	-0.117	0.243
B_THR109	-0.017	-0.004	-0.013	-0.039	-0.001	-0.039	-0.017
B_PHE110	0.000	0.000	0.000	0.000	0.000	0.000	0.000
B_ILE112	0.004	0.000	0.004	0.000	0.000	0.000	0.000
B_ASN113	0.096	-0.029	0.126	-0.167	-0.006	-0.160	-0.218
B_VAL114	0.000	0.000	0.000	0.000	0.000	0.000	0.000
B_SER116	0.000	0.000	0.000	0.000	0.000	0.000	0.000
B_ASN138	0.025	0.000	0.025	-0.014	0.000	-0.014	-0.054
B_LEU139	0.050	-0.042	0.092	-0.051	-0.031	-0.020	-0.134
B_THR140	-0.075	-0.272	0.192	-0.171	-0.255	0.083	-0.285
B_SER141	-9.109	-3.280	-5.824	-8.796	-5.208	-3.588	0.473
B_THR142	-4.435	-4.012	-0.418	-4.171	-4.015	-0.156	1.255
B_THR143	-9.899	-9.029	-0.866	-10.435	-10.468	0.033	0.096
B_TYR144	0.063	-0.188	0.251	-0.363	-0.214	-0.148	-0.071
B_TRP145	-0.464	-0.126	-0.339	-0.431	-0.149	-0.282	0.276
B_LEU146	-2.887	-2.858	-0.029	-1.922	-1.979	0.057	4.079
B_ILE148	-4.828	-4.908	0.079	-5.330	-5.401	0.072	-1.100
B_GLU149	0.142	-0.088	0.230	-0.422	-0.158	-0.264	0.054
B_ALA150	-0.054	-0.050	-0.004	0.000	-0.088	0.088	-0.046
B_TYR151	-5.489	-5.263	-0.226	-4.210	-4.072	-0.138	-1.247
B_THR152	0.172	-0.008	0.180	0.308	-0.016	0.324	0.205
B_HIS153	0.259	-0.029	0.289	-0.451	-0.019	-0.432	-0.602

B_TYR154	-13.749	-6.640	-7.113	-10.235	-6.341	-3.895	0.222
B_ILE155	-0.276	-0.402	0.126	-0.566	-0.633	0.067	-0.456
B_SER156	-0.067	-0.004	-0.063	0.080	-0.003	0.083	-0.079
B_THR157	0.109	-0.021	0.130	-0.086	-0.007	-0.080	0.167
B_LYS158	-1.443	-0.611	-0.833	-0.913	-0.403	-0.510	0.243
B_ALA159	0.192	-0.004	0.197	0.140	-0.002	0.142	-0.046
B_ALA160	0.000	0.000	0.000	0.000	0.000	0.000	0.000
B_ASN161	-0.431	-0.008	-0.427	0.085	-0.004	0.089	0.406
B_ILE162	-0.038	0.000	-0.033	-0.022	-0.002	-0.020	0.029
B_ALA181	0.000	0.000	0.000	0.002	0.000	0.002	0.000
B_ILE182	-0.331	-0.017	-0.318	-0.146	-0.025	-0.121	0.088
B_ALA183	-0.402	-0.151	-0.251	-0.318	-0.214	-0.104	0.075
B_PRO184	-1.331	-1.402	0.071	-2.927	-2.605	-0.322	-0.849
B_SER185	-3.598	-3.640	0.042	-4.264	-4.316	0.052	1.954
B_LEU186	-13.891	-13.895	0.000	-13.332	-13.315	-0.017	4.481
B_VAL187	-0.845	-0.828	-0.017	-0.746	-0.721	-0.026	0.724
B_THR189	-0.351	-0.096	-0.255	-0.071	-0.036	-0.035	0.234
B_THR191	-0.255	-0.113	-0.142	-0.162	-0.029	-0.133	0.339
B_THR192	-1.201	-1.251	0.050	-0.602	-0.545	-0.057	1.753
B_SER195	0.356	-0.222	0.577	-0.178	-0.166	-0.012	-0.477
B_LEU197	-10.042	-10.188	0.146	-8.315	-8.266	-0.048	0.757
B_PHE201	-0.519	-0.310	-0.213	-0.510	-0.351	-0.158	-0.142
B_LEU204	-2.728	-2.820	0.088	-2.015	-2.371	0.356	2.778
B_GLN215	0.134	-0.126	0.259	-0.428	-0.129	-0.298	-0.368
B_LEU220	-0.021	-0.017	-0.008	-0.051	-0.023	-0.027	-0.029
B_THR239	0.000	0.000	0.000	0.000	-0.001	0.000	-0.004
B_ALA241	0.172	-0.004	0.176	-0.010	-0.015	0.005	-0.138
B_VAL242	-0.159	-0.021	-0.142	0.259	-0.034	0.293	0.205
B_ASP243	0.180	-0.142	0.322	-1.078	-0.171	-0.907	-1.636
B_MET246	-1.021	-0.929	-0.092	-0.565	-0.778	0.213	0.582
B_VAL247	-0.054	-0.075	0.021	-0.185	-0.091	-0.094	-0.105
B_NAD256	-12.862	-12.376	-0.490	-5.658	-6.322	0.664	11.033
SUM	-109.529	-95.345	-14.184	-91.215	-82.019	-9.196	

Table S6 Calculated interaction energies of 4′-aminoacetophenone in PEDH active site in Prelog and anti-Prelog positions.

Residue	Interaction Energy (kJ/mol)	VDW Interaction Energy (kJ/mol)	Electrostatic Interaction Energy (kJ/mol)	Interaction Energy (kJ/mol)	VDW Interaction Energy (kJ/mol)	Electrostatic Interaction Energy (kJ/mol)	Δ IE (AntiP-P) (kJ/mol)
	**Prelog (pro(*S*))**			**Anti-Prelog (pro(*R*))**			
B_GLY14	0.000	0.000	0.000	0.000	0.000	0.000	0.000
B_GLY18	0.025	0.000	0.025	0.000	0.000	0.000	-0.025
B_ILE19	-0.092	-0.105	0.013	-0.159	-0.067	-0.092	-0.063
B_GLY20	0.017	0.000	0.017	0.000	0.000	0.000	-0.017
B_ASN88	0.000	0.000	0.000	0.000	0.000	0.000	0.000
B_ASN89	0.356	-0.008	0.364	0.640	0.000	0.640	0.285
B_ALA90	-0.092	0.000	-0.092	0.000	0.000	0.000	0.092
B_GLY91	0.033	-0.050	0.079	0.025	-0.008	0.029	-0.008
B_ILE92	-0.088	-0.234	0.146	0.008	-0.063	0.071	0.096
B_TYR93	-8.531	-8.058	-0.469	-1.833	-1.586	-0.247	6.699
B_PRO94	-0.172	-0.155	-0.013	-0.176	-0.084	-0.092	-0.004
B_LEU95	-0.021	-0.238	0.213	-0.109	-0.205	0.092	-0.088
B_ILE96	-0.038	-0.004	-0.038	-0.126	-0.008	-0.117	-0.084
B_THR109	-0.017	-0.004	-0.013	-0.038	0.000	-0.038	-0.021
B_PHE110	0.000	0.000	0.000	0.000	0.000	0.000	0.000
B_ILE112	0.004	0.000	0.004	0.000	0.000	0.000	-0.004
B_ASN113	0.096	-0.029	0.126	-0.167	-0.008	-0.159	-0.264
B_VAL114	0.000	0.000	0.000	0.000	0.000	0.000	0.000
B_SER116	0.000	0.000	0.000	0.000	0.000	0.000	0.000
B_ASN138	0.025	0.000	0.025	-0.013	0.000	-0.013	-0.042
B_LEU139	0.050	-0.042	0.092	-0.050	-0.029	-0.021	-0.100
B_THR140	-0.075	-0.272	0.192	-0.172	-0.255	0.084	-0.096
B_SER141	-9.109	-3.280	-5.824	-8.795	-5.209	-3.590	0.310
B_THR142	-4.435	-4.012	-0.418	-4.171	-4.017	-0.155	0.264
B_THR143	-9.899	-9.029	-0.866	-10.435	-10.468	0.033	-0.540
B_TYR144	0.063	-0.188	0.251	-0.364	-0.213	-0.146	-0.427
B_TRP145	-0.464	-0.126	-0.339	-0.431	-0.151	-0.280	0.033
B_LEU146	-2.887	-2.858	-0.029	-1.920	-1.979	0.059	0.967
B_ILE148	-4.828	-4.908	0.079	-5.330	-5.402	0.071	-0.502
B_GLU149	0.142	-0.088	0.230	-0.423	-0.159	-0.264	-0.565
B_ALA150	-0.054	-0.050	-0.004	0.000	-0.088	0.088	0.054
B_TYR151	-5.489	-5.263	-0.226	-4.209	-4.071	-0.138	1.280
B_THR152	0.172	-0.008	0.180	0.310	-0.017	0.322	0.138
B_HIS153	0.259	-0.029	0.289	-0.452	-0.021	-0.431	-0.707

B_TYR154	-13.749	-6.640	-7.113	-10.234	-6.339	-3.895	3.515
B_ILE155	-0.276	-0.402	0.126	-0.565	-0.632	0.067	-0.289
B_SER156	-0.067	-0.004	-0.063	0.079	-0.004	0.084	0.146
B_THR157	0.109	-0.021	0.130	-0.088	-0.008	-0.079	-0.197
B_LYS158	-1.443	-0.611	-0.833	-0.912	-0.402	-0.510	0.531
B_ALA159	0.192	-0.004	0.197	0.138	0.000	0.142	-0.054
B_ALA160	0.000	0.000	0.000	0.000	0.000	0.000	0.000
B_ASN161	-0.431	-0.008	-0.427	0.084	-0.004	0.088	0.519
B_ILE162	-0.038	0.000	-0.033	-0.021	-0.004	-0.021	0.013
B_ALA181	0.000	0.000	0.000	0.000	0.000	0.000	0.000
B_ILE182	-0.331	-0.017	-0.318	-0.146	-0.025	-0.121	0.184
B_ALA183	-0.402	-0.151	-0.251	-0.318	-0.213	-0.105	0.084
B_PRO184	-1.331	-1.402	0.071	-2.929	-2.607	-0.322	-1.594
B_SER185	-3.598	-3.640	0.042	-4.263	-4.318	0.050	-0.665
B_LEU186	-13.891	-13.895	0.000	-13.334	-13.313	-0.017	0.561
B_VAL187	-0.845	-0.828	-0.017	-0.745	-0.720	-0.025	0.096
B_THR189	-0.351	-0.096	-0.255	-0.071	-0.038	-0.033	0.280
B_THR191	-0.255	-0.113	-0.142	-0.163	-0.029	-0.134	0.092
B_THR192	-1.201	-1.251	0.050	-0.602	-0.544	-0.059	0.598
B_SER195	0.356	-0.222	0.577	-0.180	-0.167	-0.013	-0.536
B_LEU197	-10.042	-10.188	0.146	-8.314	-8.268	-0.050	1.724
B_PHE201	-0.519	-0.310	-0.213	-0.510	-0.351	-0.159	0.008
B_LEU204	-2.728	-2.820	0.088	-2.017	-2.372	0.356	0.715
B_GLN215	0.134	-0.126	0.259	-0.427	-0.130	-0.297	-0.565
B_LEU220	-0.021	-0.017	-0.008	-0.050	-0.025	-0.029	-0.029
B_THR239	0.000	0.000	0.000	0.000	0.000	0.000	0.000
B_ALA241	0.172	-0.004	0.176	-0.008	-0.017	0.004	-0.180
B_VAL242	-0.159	-0.021	-0.142	0.259	-0.033	0.293	0.418
B_ASP243	0.180	-0.142	0.322	-1.079	-0.172	-0.908	-1.259
B_MET246	-1.021	-0.929	-0.092	-0.565	-0.778	0.213	0.456
B_VAL247	-0.054	-0.075	0.021	-0.184	-0.092	-0.096	-0.130
B_NAD256	-12.862	-12.376	-0.490	-5.657	-6.322	0.665	7.205
SUM	-109.529	-95.345	-14.184	-91.215	-82.019	-9.196	

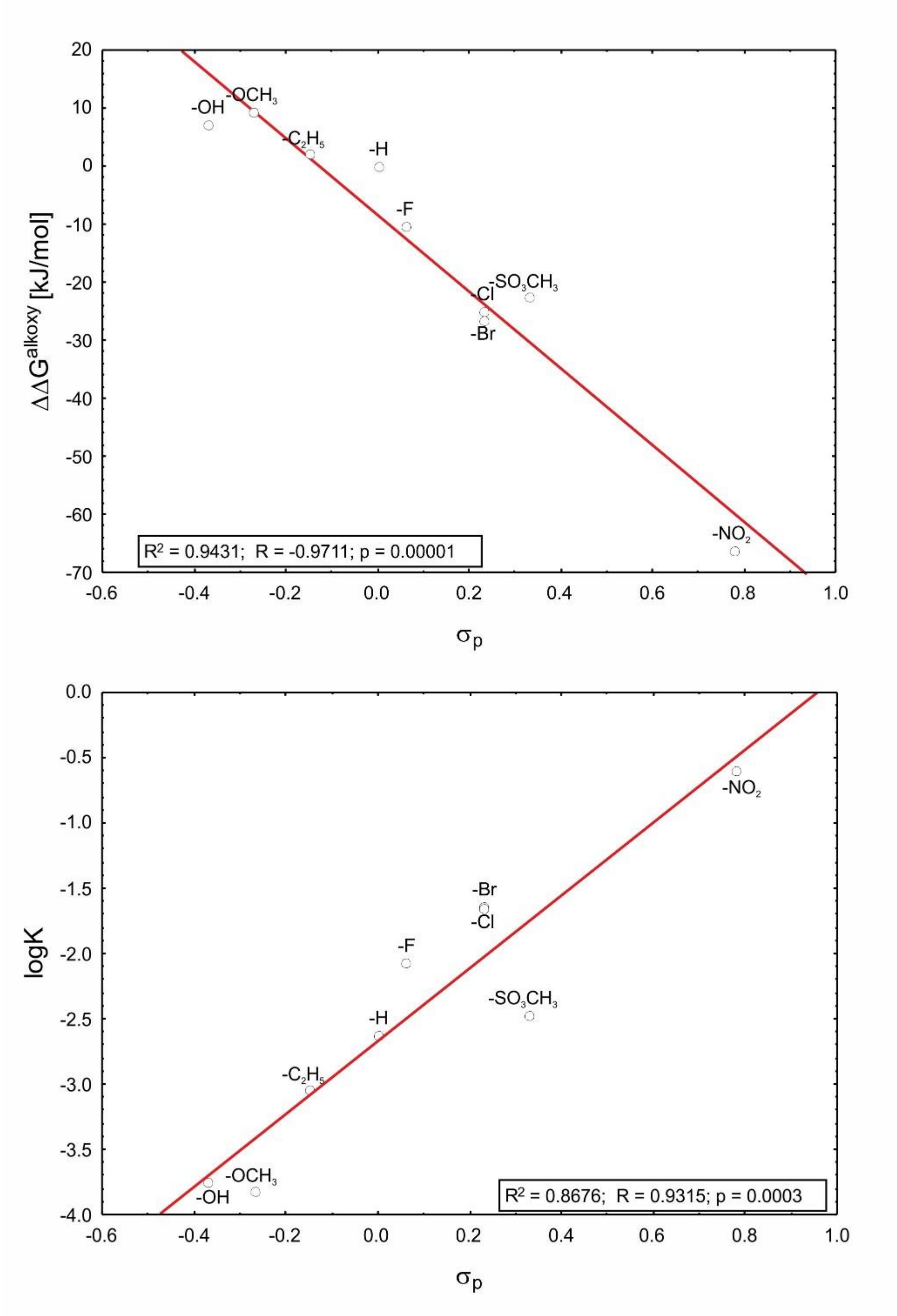

Fig. S1 Scatter plots showing linear correlations between Hammett σp values and calculated $\Delta\Delta G^{alkoxy}$ and log K descriptors of various PEDH substrates (see Table S1).

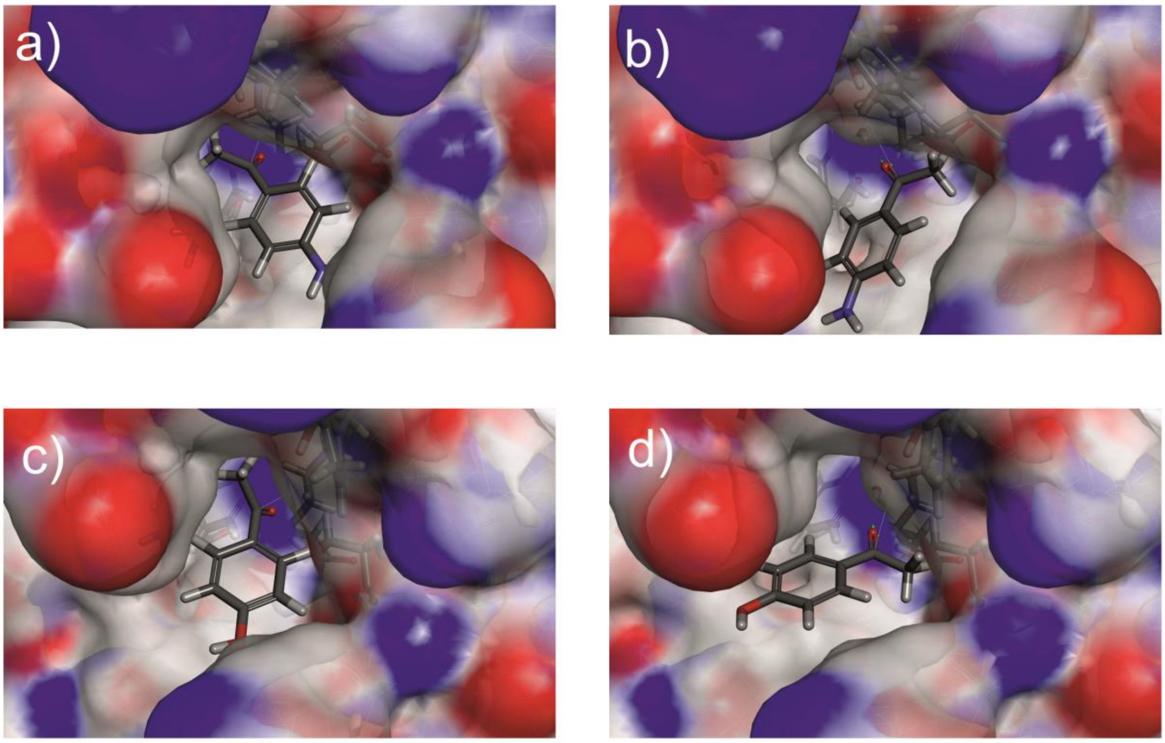

Fig. S2 Modeled ternary complexes of PEDH with bound substrate and NADH: A) Prelog orientation (Pro(*S*)) and B) anti-Prelog (Pro(*R*)) orientation of 4’-aminoacetophenone, C) Prelog orientation (Pro(*S*)) and B) anti-Prelog (Pro(*R*)) orientation of 4’-hydroxyacetophenone. The vdW surface of PEDH is colored according to the H-bond acceptor (blue) / H-bond donor (red) capabilities of the protein residues.

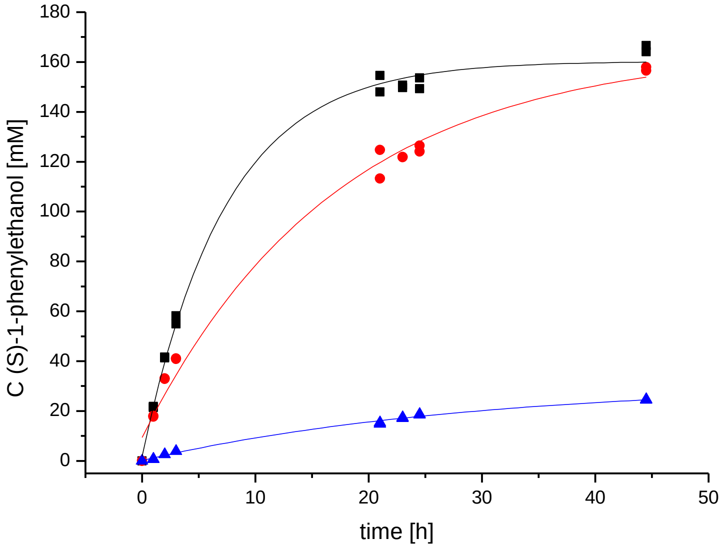

Fig. S3 The progress curves obtained for conversion of acetophenone with various concentrations of IPA: blue triangles: minimal concentration of 14% IPA, red circles: 60% IPA, black squares: 95% IPA. The reaction with 14% IPA was conducted with an initial acetophenone concentration C_0 of 100 mM while reactors with 60 and 95% IPA were started with a C_0 of 200 mM.

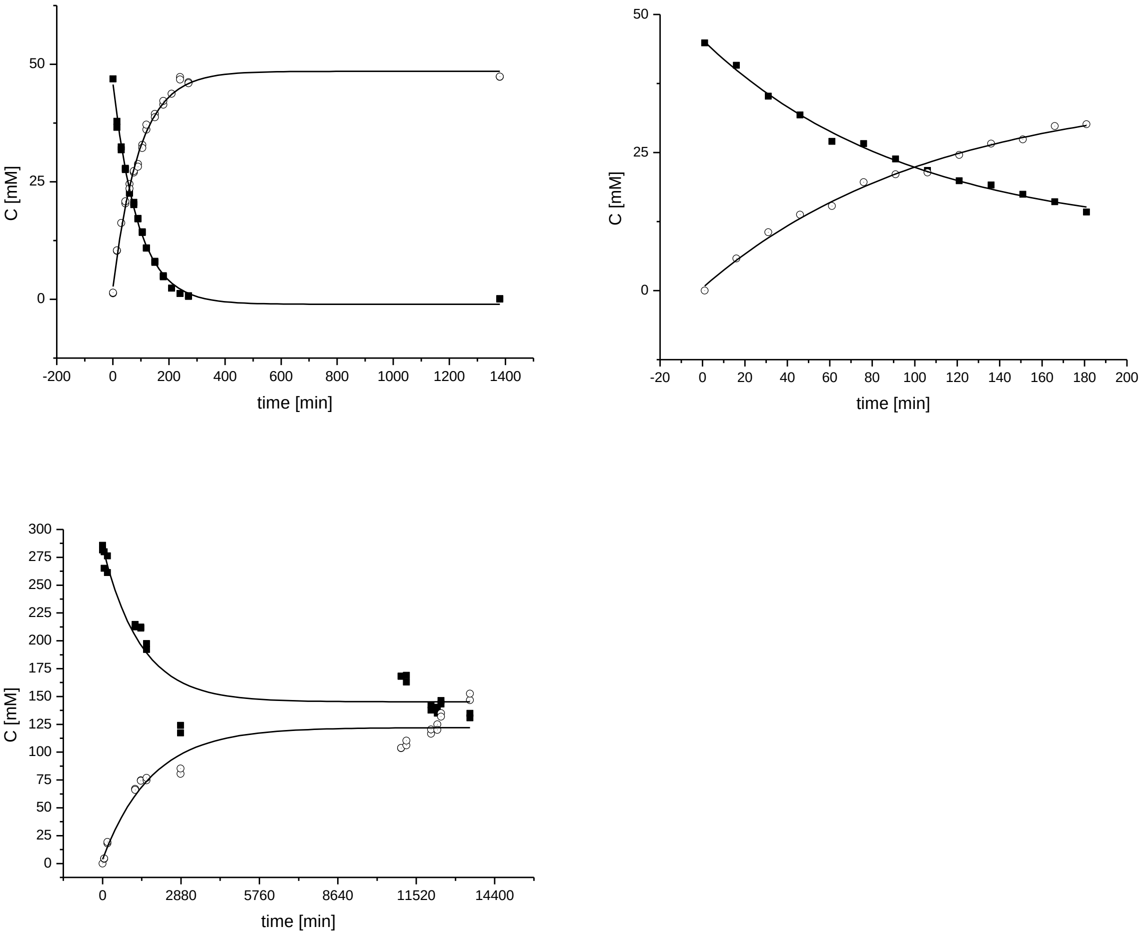

Fig. S4A Acetophenone reduction progress curves (squares - substrate, circles – product). Top: reaction with C_0= 47 mM and 45 mM, bottom: reaction with C_0= 286 mM.

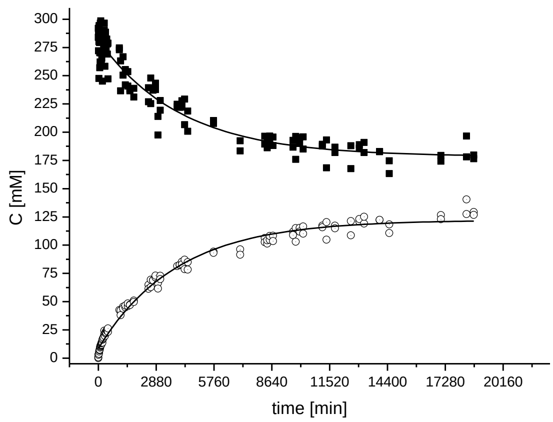

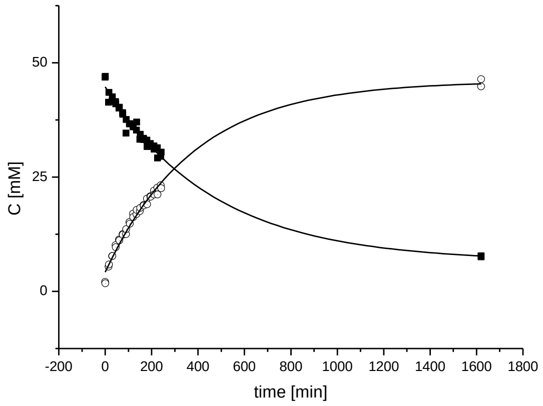

Fig. S4B *p*-ethylacetophenone reduction progress curve (squares - substrate, circles – product); left C_0= 47 mM, right: reaction with C_0= 292 mM.

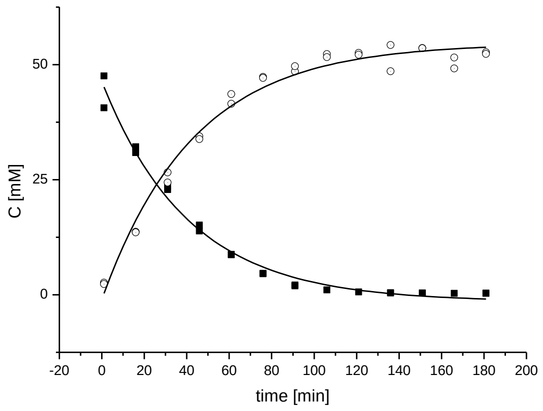

Fig. S4C 4’-acetylphenyl methanesulfonate reduction progress curve (squares - substrate, circles – product). C_0 = 52 mM.

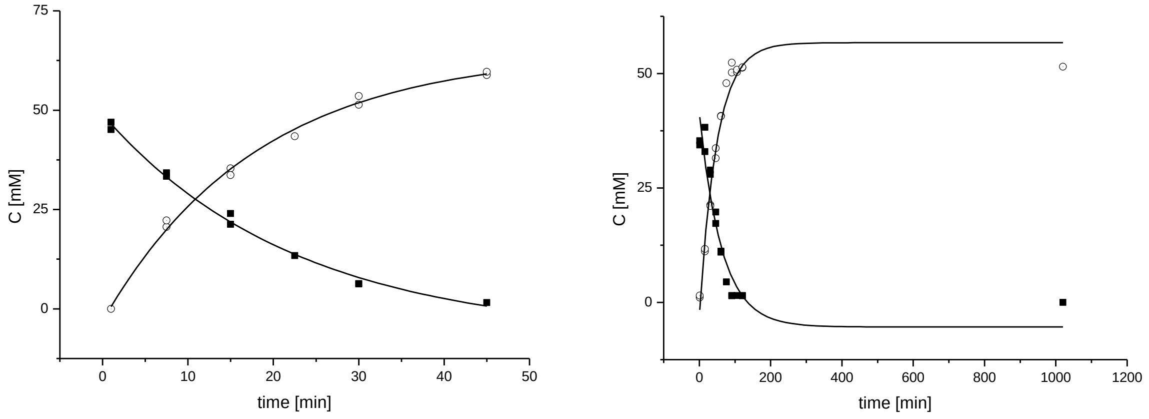

Fig. S4D *p*-nitroacetophenone reduction progress curves (squares - substrate, circles – product). C_0 = 55 and 49 mM.

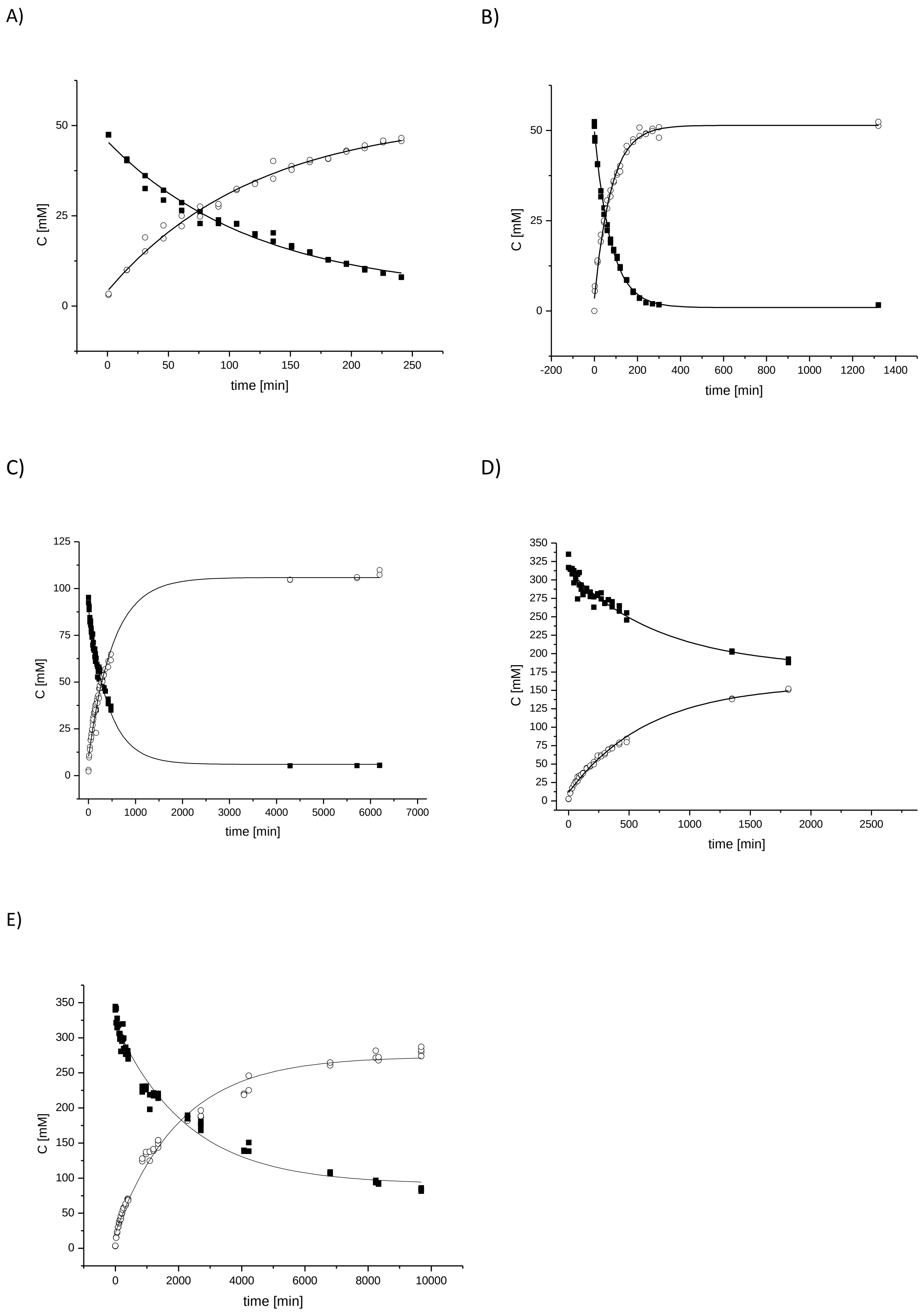

Fig. S4E *p*-Fluoroacetophenone reduction progress curves (squares - substrate, circles – product). Reactions with C_0 =: A) 47 mM , B) 50 mM ,C) 107 mM, D) 335 mM and E) 356 mM

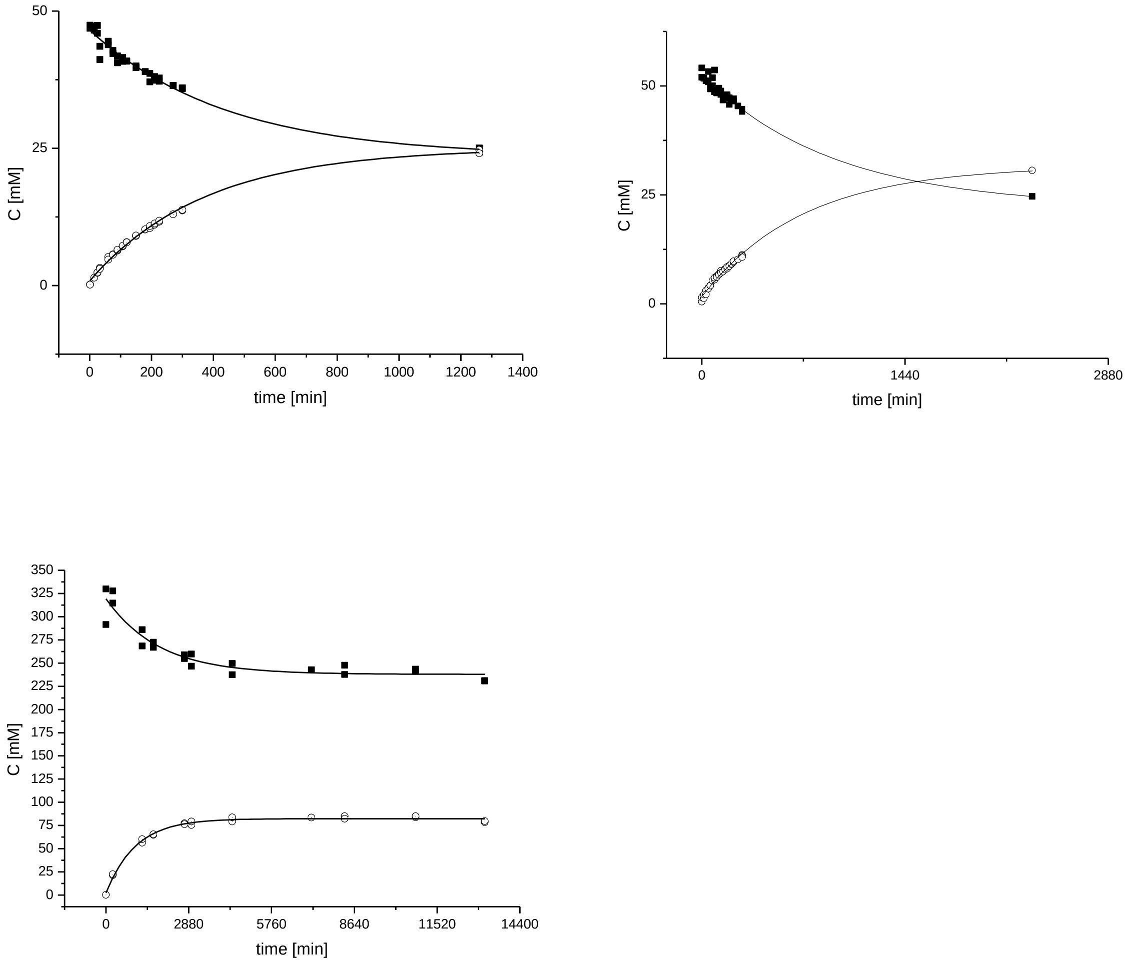

Fig. S4F *p*-Hydroxyacetophenone reduction progress curves (squares - substrate, circles – product). Top: reactions with C_0 = 47 and 55 mM, bottom C_0 = 330 mM.

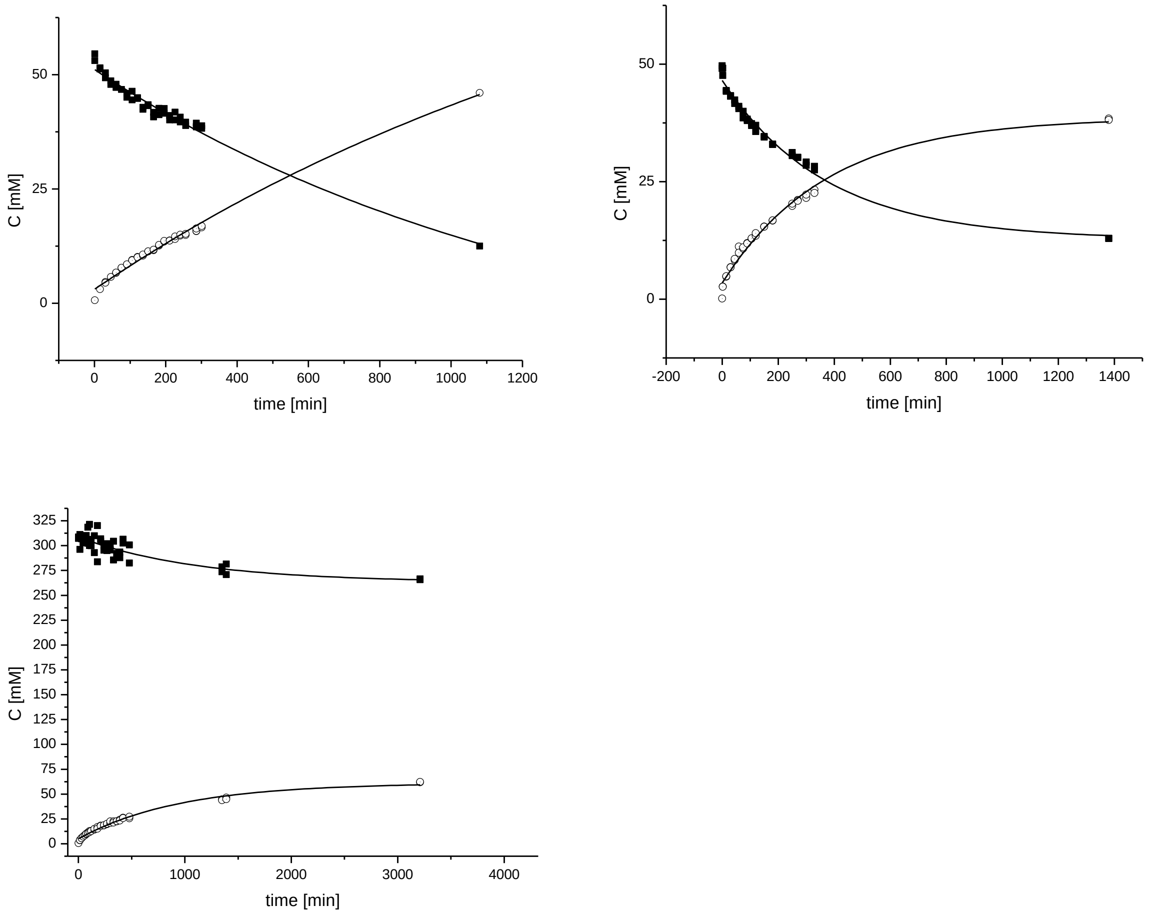

Fig. S4G *p*-methoxyacetophenone reduction progress curves (squares - substrate, circles – product).). Top: reactions with C_0 = 55 and 49 mM, bottom C_0 = 309 mM.

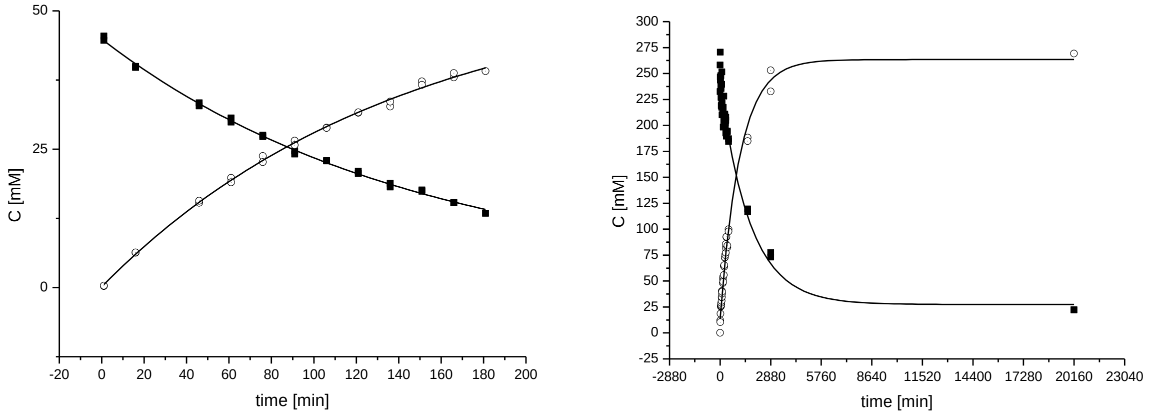

Fig. S4H *p*-Chloroacetophenone reduction progress curves (squares - substrate, circles – product) Left: reaction with C_0= 45 mM, right: reaction with C_0= 273 mM.

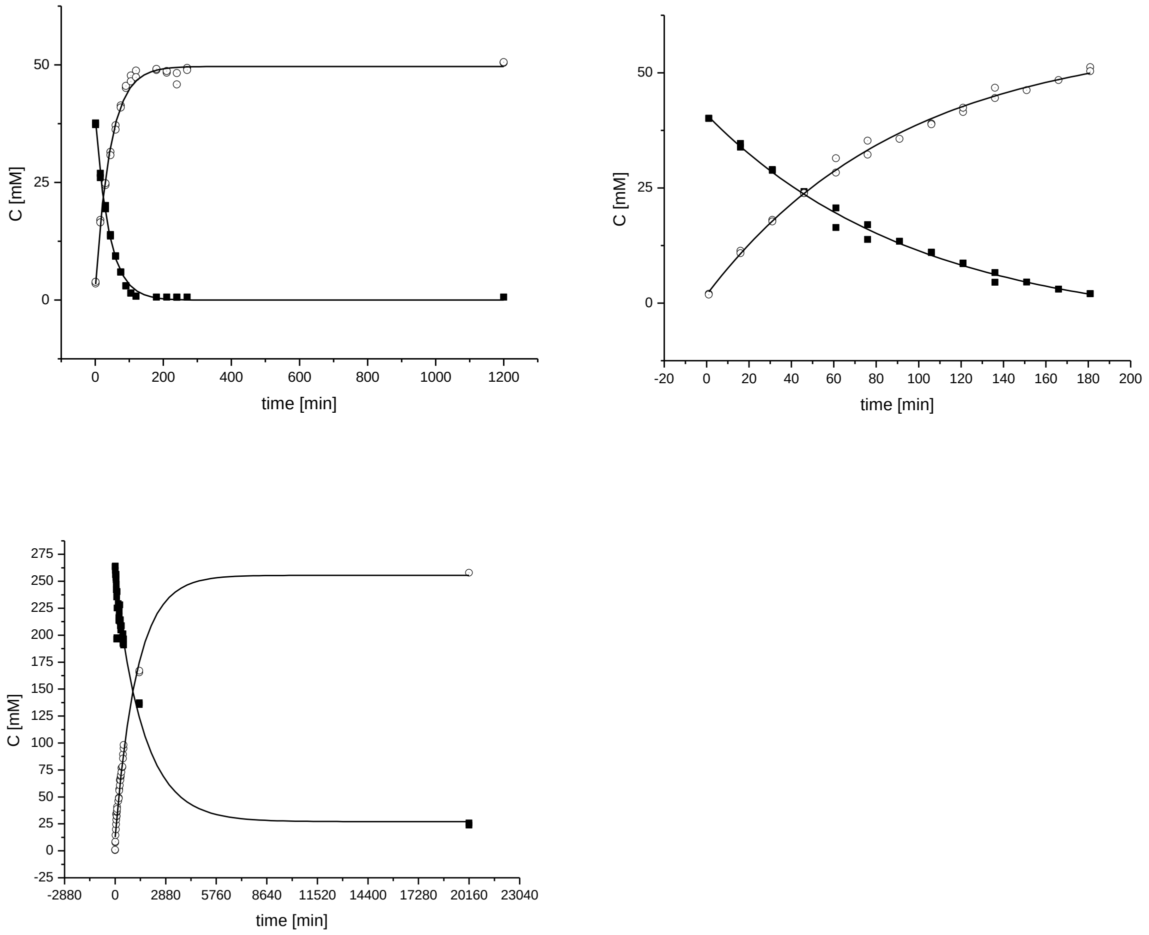

Fig. S4I *p*-Bromoacetophenone reduction progress curves (squares - substrate, circles – product). Top: reactions with C_0= 52 and 50 mM, bottom: reaction with C_0= 275 mM.

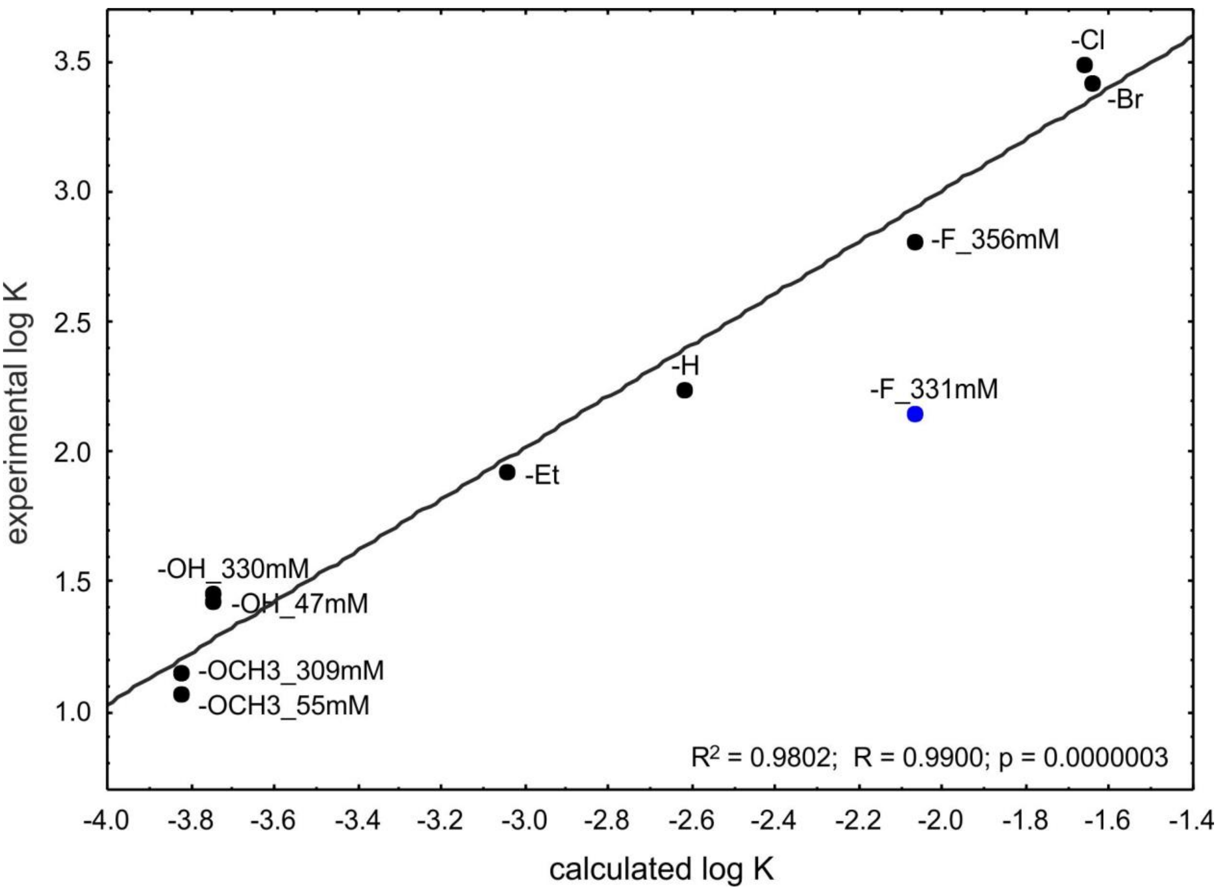

Figure S5. Correlation plot of calculated log K with log K estimated from batch reactor tests. The indexes at the point description provide initial concentration of the reactor in case of multiple runs for a particular substrate. The reactor with p-F-acetophenone (C_0=331 mM, blue circle) was excluded from the analysis as the reaction progress was not followed until equilibrium which results with underestimation of the experimental log K.

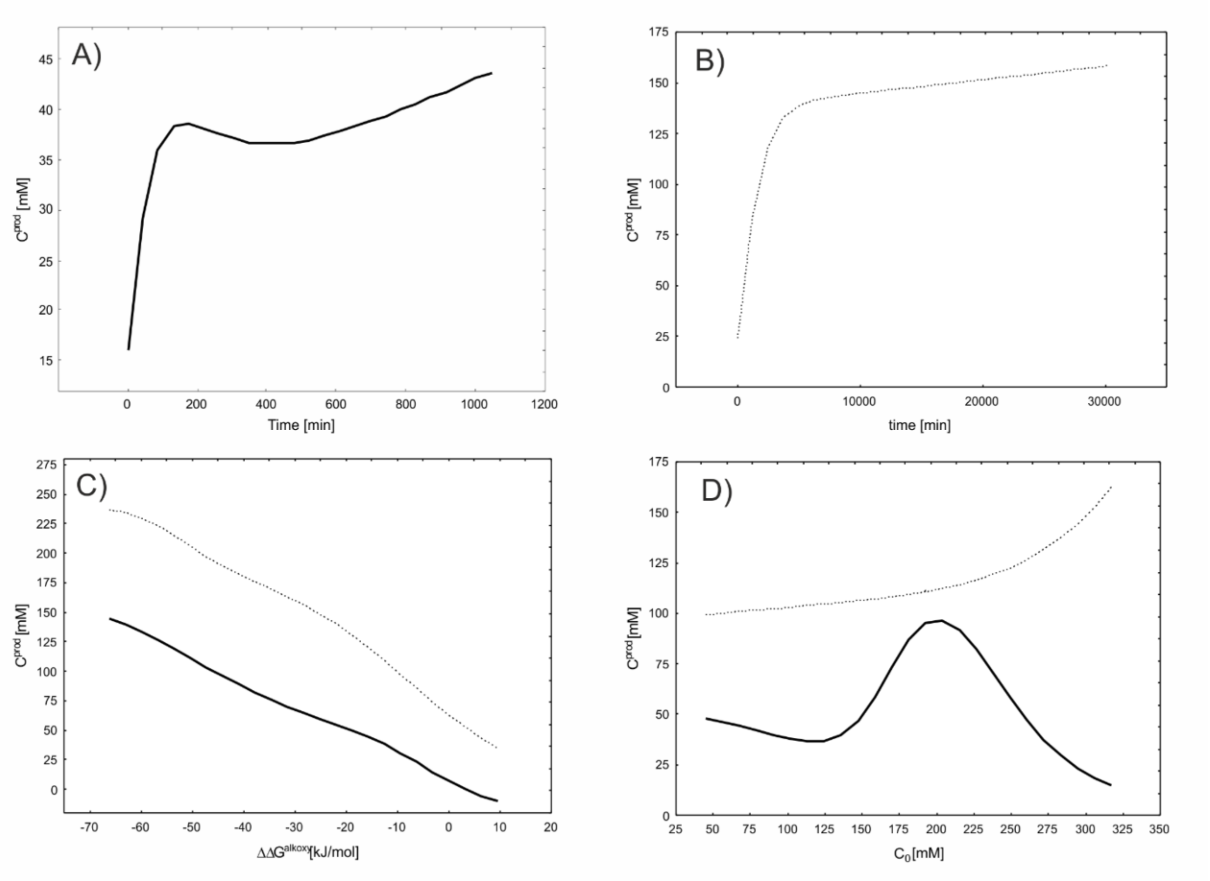

Fig. S6 Response curves for model 1 (dots) and model 2 (solid line) showing dependence of product concentration from: A) and B) reaction time, C) $\Delta\Delta G^{alkoxy}$ and D) initial substrate concentration C_0. The response curves are generated as a 2D projection of model behavior provided all the other input variables are constant at their average values for the given dataset.

Generation of the points for ANN modeling

In order to avoid modeling artifacts derived from uneven data collection during long-lasting experiments, the experimental product concentrations were fitted with the equation $C(t)=C_0+A_1e^{(-t/k_1)}$ and new concentration *vs.* time points were generated depending on the total time of the conversion experiment (160 and 80 points for reaction with 300 and 50 mM substrate initial concentration, respectively). For all 7 batch reactions with app. 300 mM initial substrate concentrations, a uniform duration of the reaction (30240 min., 504 h) was generated with mono-exponential equations that were fitted to experimental data. This selected time corresponded to the duration of the longest-lasting reaction recorded in the lab. For each progress curve 160 data points were generated. The initial 120 of these points were placed in even distribution from the start of the reaction curve until the reaction reached the saturation plateau, whereas another 40 data points were used to indicate the saturated part of the progress curve up to the time limit at 504 h . An analogous procedure was used for modeling of 15 progress curves of reactions with initial concentrations of 50 mM where the uniform time of reaction was set to 885 min. The initial part of these curves was indicated by 60 evenly distributed points, while the saturation plateau was indicated by 20 more data points.

The applied procedure yielded 2320 individual model runs (1120 for reactions with C_0 = 300 mM and 1200 with C_0 = 50 mM). These were randomly divided into training, validation and test subsets in which a 2:1:1 ratio was performed.

Parameters of neural models

Each of neural models utilized the same architecture, i.e. 3 input neurons, 5 neurons in a hidden layer and one output neuron (Fig. S7). Each of the input neurons (1.1-1.3) introduced the input variable into the model and performed a minmax normalization of its value to the 0-1 range. Each of the input neurons transmits its normalized value to every hidden neuron (2.1-2.5). In turn each of the hidden neurons conducts signal aggregation by means of linear combination of inputs $\varphi = \sum_{i=0}^{n} w_i \, x_i{}^k + \theta$. The applied constants (weights and threshold θ) of the aggregation functions φ are collected in Table S8 for model 1 and S9 for model 2. The result of signal aggregation was used as an argument of a hyperbolic activation function $\psi = \frac{e^x - e^{-x}}{e^x + e^{-x}}$ yielding results in the range of (-1,+1). Results (activation level) of each neuron of the hidden layer were subsequently transmitted to the output neuron (3.1)

and subjected to another linear aggregation function φ (consents Table S7 for model 1 and S8 form model 2) and logistic activation function $\vartheta = \frac{1}{1-e^{-x}}$..

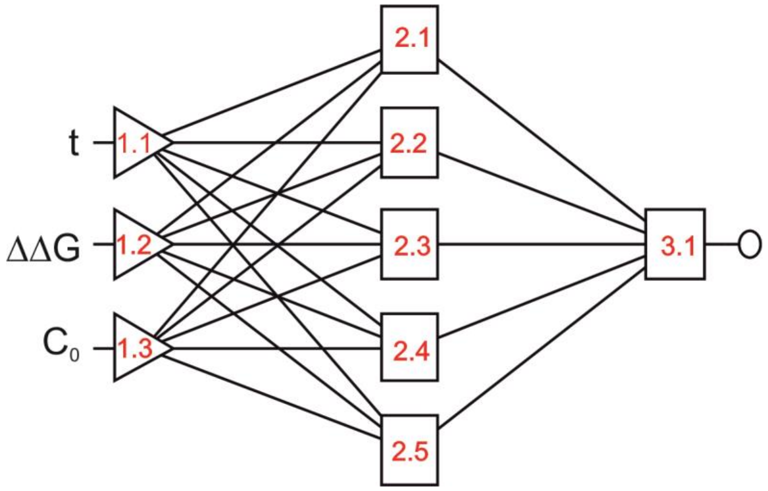

Table S7. Neural weights and threshold constants of aggregation functions of ANN model 1.

Neuron number	**2.1**	**2.2**	**2.3**	**2.4**	**2.5**	**3.1**
**Threshold θ**	-0.8501	0.844192	-0.84359	0.23543	-0.340169	1.22069
**1.1**	-12.1457	-0.080186	0.02886	-1.28608	-0.116256	
**1.2**	-2.1693	1.374649	-1.23743	-4.45710	0.465425	
**1.3**	0.4429	-0.025585	0.29380	3.02404	0.061103	
**2.1**						-3.39422
**2.2**						-2.06977
**2.3**						1.59749
**2.4**						1.60051
**2.5**						-0.53216

Table S8. Neural weights and threshold constants of aggregation functions of ANN model 2.

Neuron number	2.1	2.2	2.3	2.4	2.5	3.1
**Threshold θ**	-0.468157	0.55927	4.775499	0.56061	3.89830	0.67296
1.1	9.192675	-2.47865	-0.547622	-1.13774	2.50739	
1.2	0.772571	-2.39801	4.323864	-1.38222	-3.24668	
1.3	-0.138809	6.63746	3.494382	2.67944	2.50640	
2.1						4.02915
2.2						2.44843
2.3						-2.00548
**2.4**						-1.26648
**2.5**						4.68064

Table S9 Results of calculation of binding energies. BE – binding energy calculated as a difference of the energy of the protein-ligand complex versus the sum of energies of ligand and protein alone. TBE – total binding energy is BE corrected for ligand conformational energy fractions. TBE + ∆S - total binding energy corrected for entropic energy fractions (entropy of binding calculated as a difference of entropy of the protein-ligand complex versus the sum of entropies of free protein and ligand).

No	Substarte / pose	BE kJ/mol	TBE kJ/mol	TBE + ∆S kJ/mol.	Ligand Energy (kJ/mol)	Protein Energy (kJ/mol)	Complex Energy (kJ/mol)	Entropic Energy (kJ/mol)	Ligand Conformational Energy (kJ/mol)	Ligand Conformational Entropy (kJ/mol-K)
1	Acetophenone Prelog	-101.5	-100.0	-29.3	23.5	-6775.2	-6853.1	70.5	1.7	1.7
1	Acetophenone anti-Prelog	-78.3	-76.6	-6.3	11.3	-6775.2	-6842.2	70.4	1.7	1.7
7	4’-acetylbiphenyl	-117.6	-112.5	-36.0	51.8	-6775.2	-6841.0	76.4	5.1	5.1
21	4’-aminoacetophenone Prelog	-116.1	-114.6	-42.7	13.3	-6775.2	-6878.0	71.8	1.7	1.7
21	4’-aminoacetophenone anti-Prelog	-82.6	-80.8	-9.2	9.5	-6775.2	-6848.2	71.8	1.7	1.7
22	4’-hydroxyacetophenone Prelog	-118.3	-116.7	-44.8	17.7	-6775.2	-6875.7	71.8	1.7	1.7
22	4’-hydroxyacetophenone anti-Prelog	-86.6	-84.9	-13.0	13.3	-6775.2	-6848.4	71.8	1.7	1.7
28	2,4’-dichloroacetophenone	-118.4	-114.2	-38.9	21.5	-6775.2	-6872.0	75.6	4.1	4.1
29	2,2,2-trifluoroacetophenone	-120.5	-112.5	-38.5	34.1	-6775.2	-6861.6	74.0	7.9	7.9
43	methyl 4-fluorobenzoylacetate	-194.3	-184.1	-107.9	15.4	-6775.2	-6954.0	76.3	10.3	6.6
